# Gene Signatures of NEUROGENIN3+ Endocrine Progenitor Cells in the Human Pancreas

**DOI:** 10.3389/fendo.2021.736286

**Published:** 2021-09-08

**Authors:** Hyo Jeong Yong, Gengqiang Xie, Chengyang Liu, Wei Wang, Ali Naji, Jerome Irianto, Yue J. Wang

**Affiliations:** ^1^Department of Biomedical Sciences, College of Medicine, Florida State University, Tallahassee, FL, United States; ^2^Department of Surgery, Hospital of the University of Pennsylvania, Philadelphia, PA, United States

**Keywords:** NEUROG3, endocrine progenitor, epsilon cells, human pancreas, single-cell RNA-seq, data integration

## Abstract

NEUROGENIN3+ (NEUROG3+) cells are considered to be pancreatic endocrine progenitors. Our current knowledge on the molecular program of NEUROG3+ cells in humans is largely extrapolated from studies in mice. We hypothesized that single-cell RNA-seq enables in-depth exploration of the rare NEUROG3+ cells directly in humans. We aligned four large single-cell RNA-seq datasets from postnatal human pancreas. Our integrated analysis revealed 10 NEUROG3+ epithelial cells from a total of 11,174 pancreatic cells. Noticeably, human NEUROG3+ cells clustered with mature pancreatic cells and epsilon cells displayed the highest frequency of NEUROG3 positivity. We confirmed the co-expression of NEUROG3 with endocrine markers and the high percentage of NEUROG3+ cells among epsilon cells at the protein level based on immunostaining on pancreatic tissue sections. We further identified unique genetic signatures of the NEUROG3+ cells. Regulatory network inference revealed novel transcription factors including Prospero homeobox protein 1 (PROX1) may act jointly with NEUROG3. As NEUROG3 plays a central role in endocrine differentiation, knowledge gained from our study will accelerate the development of beta cell regeneration therapies to treat diabetes.

## Introduction

*Neurogenin 3* (*Neurog3*) encodes a basic helix-loop-helix transcription factor considered to be the master regulator of pancreatic endocrine differentiation. The crucial role of *Neurog3* has been established in mice. *Neurog3* deficient mice do not have any pancreatic endocrine cells, develop diabetes, and die shortly after birth (1). Conversely, overexpression of *Neurog3* in the non-endocrine epithelium converts these cells to endocrine cell fates (1–4). In the developing pancreas, NEUROG3 expression is biphasic (5). It is first detected at embryonic day (E) 8.5 during the primary transition and the expression declines to near zero at E11.5. NEUROG3 expression becomes detectable again during the secondary transition and peaks at E15.5 (5). NEUROG3+ cells are considered multipotent endocrine progenitor cells: They never co-express mature hormone markers; instead, they have the potential to differentiate into different endocrine lineages *in vivo* and *in vitro* (6). In the adult mouse pancreas, under normal homeostasis, a small number of NEUROG3+ cells can be detected. Lineage tracing experiments indicate that these NEUROG3+ cells are residual islet progenitors (6). Upon pancreatic tissue injury, several studies suggest that NEUROG3 is upregulated and NEUROG3+ cells contribute to beta cell regeneration in adults (7–9). Recent studies indicate that lineage bias among the NEUROG3+ population exists and single NEUROG3+ cells are predetermined to differentiate into one specific endocrine cell type through epigenetic regulation (10, 11).

In humans, the significant role of NEUROG3 in pancreatic endocrine differentiation has been revealed in genetics studies, as patients carrying *NEUROG3* mutations often develop neonatal diabetes (12–15). The importance of NEUROG3 has been further confirmed *in vitro* where it is absolutely required for the derivation of mature human beta cells from pluripotent stem cells (16). In fact, the NEUROG3+ stage is an essential stage in all the *in vitro* differentiation protocols (17–19). Contrary to mice where two waves of *Neurog3* expression are observed, human NEUROG3 is expressed in a single wave during embryonic development (20, 21). The expression of NEUROG3 gradually reduces in abundance as the development progresses (20–22). It remains controversial as to whether NEUROG3 is expressed in the post-development human pancreas (20, 23–25), possibly reflecting the detection limit of immunolabeling approaches and the sensitivities as well as specificities of antibodies utilized in some of the studies (26).

Because of the central role of NEUROG3 in endocrine differentiation, understanding the regulatory network of NEUROG3, as well as the molecular characteristics of NEUROG3+ cells in humans, has large implications for diabetes treatment. For instance, the features of endogenous NEUROG3+ cells can serve as a benchmark against which the authenticity of NEUROG3+ cells derived *in vitro* can be compared. Knowledge gained from such comparison can help to further optimize the stepwise differentiation protocol to generate functional beta cells from pluripotent stem cells. Furthermore, the existence of NEUROG3+ cells in the postnatal pancreata would raise the possibility of *in vivo* modulating these cells for beta cell regenerative therapy.

The ultra-low abundance of NEURGO3+ cells in the postnatal human pancreas renders it challenging to capture, let alone to investigate these cells in detail. The development and maturation of single-cell RNA-seq technology largely overcomes the limitation in the resolution of bulk assays and significantly increases the sensitivity to profile rare cell types. In this study, we integrated four single-cell RNA-seq datasets of human pancreatic endocrine cells and retrieved NEUROG3+ cells *post hoc* in a label-free manner (27–31). This strategy guarantees the utmost purity and authenticity of NEUROG3+ cells. Here, we report the unambiguous identification of NEUROG3+ cells in postnatal human pancreata. We describe the transcriptomic programs of these NEUROG3+ cells. We present novel putative transcription factors that are likely to cooperate with NEUROG3. We also outline the similarities of these postnatal NEUROG3+ cells with NEUROG3+ cells in the fetal pancreas.

Our work, to our knowledge, is the first to delineate the transcriptomic landscape of the naturally occurring NEUROG3+ cells in humans. Our study unveils the regulatory principles of these cells and provides a resource for the research community to further explore in order to identify, isolate, and program NEUROG3+ cells in humans. The insight will help to promote the development of beta cell regenerative medicine for treating diabetes.

## Materials and Methods

### Single-Cell RNA-Seq Datasets

Raw FASTQ files of the single-cell RNA-seq studies were downloaded from publicly available data depositories as following: Enge (GSE81547), Segerstolpe (E-MTAB-5061), Wang_C1 (GSE83139 and GSE154126), and Wang_C1HT (https://hpap.pmacs.upenn.edu/). Raw reads were aligned to the GRCh38 genome assembly with STAR (version 020201) using default parameters (32). Aligned reads were visualized with the Integrative Genomics Viewer (IGV 2.8.0) (33).

Single-nucleus RNA-seq dataset was downloaded as a Seurat object from http://singlecell.charite.de/pancreas/.

### Data Integration and the Identification of NEUROG3+ Cells

Single cells from different datasets were integrated with the SCTransform integration pipeline with Seurat V3.2.0 (34). A resolution of 0.4 was used for cell clustering based on the stability of the resulting clusters analyzed with clustree (35). Uniform Manifold Approximation and Projection (UMAP) (36) was used to reduce the data dimensions and visualize the cells.

The gene expression values stored in the “RNA” assay in the integrated Seurat object were used to visualize marker gene expression as well as to select NEUROG3+ cells. Cell type classification was performed based on the high expression of marker genes in different clusters. Specifically, Insulin (INS) for beta cells, Glucagon (GCG) for alpha cells, Somatostatin (SST) for delta cells, Ghrelin (GHRL) for epsilon cells, Pancreatic polypeptide (PPY) for pancreatic polypeptide cells, Keratin18 (KRT18) for ductal cells, Serine protease 1 (PRSS1) for acinar cells, Secreted protein acidic and cysteine rich (SPARC) for fibroblasts, Von willebrand factor (VWF) for endothelial cells, and Lysosomal protein transmembrane 5 for immune cells. One cluster with high expression of multiple endocrine markers was denoted as doublets.

Twelve NEUROG3+ cells were identified as cells with >0 normalized read counts aligned to the NEUROG3 transcript. Only the 10 epithelial NEUROG3+ cells were used for downstream studies.

### Differential Expression and Pathway Analysis

Limma trend (37) was used for differential expression analysis of the NEUROG3+ cells compared with other pancreatic epithelial cells excluding cells from endothelial, fibroblast, or immune lineages. To control for the batch effects and the impact of cell type heterogeneity, differential expression was performed with the following design matrix: NormalizedCounts ~ Label + Tech + CellType, where NormalizedCounts is the matrix containing logCPM of gene expression data, Tech indicates the dataset origins and CellType points to different cell type identities. Differentially expressed genes were selected with a false discovery rate of less than 5%.

### Transcriptional Network Analysis

Raw reads aligned to transcription factors were converted to log2TPM with 1 pseudocount. The top 50 transcription factors with the highest expression in the NEUROG3+ cells were selected. Hierarchical clustering was performed with Ward’s method and Pearson correlation of the average gene expressions as the distance measurement. Their interaction networks were annotated with STRING V11.0 (38) with default settings.

### Comparisons of Postnatal NEUROG3+ Cells With NEUROG3+ Cells in the Human Fetal Pancreata

For comparison with endocrine progenitors from human fetal pancreata, single-cell qPCR results were extracted from Table S3 in Ramond et al. (39). To convert normalized read counts from single-cell RNA-seq data to the same scale as the qPCR data, log2CPM values with pseudocount of 2 were calculated for each gene in each of the 10 NEUROG3+ cells. In the combined dataset, hierarchical clustering was performed with Ward’s method and Pearson correlation of the average gene expressions as the distance matrix. The original cell labels from Ramond et al. were transferred.

### Comparisons of Postnatal NEUROG3+ Cells With NEUROG3+ Cells Emerged During *In Vitro* Differentiation From Embryonic Stem Cells

For comparison with NEUROG3+ cells derived *in vitro*, the normalized count matrix containing NEUROG3+ cells collected from stage 6 day 1 differentiation stage was obtained from GSM3402517 (40). Data integration was performed with Seurat SCTransform integration pipeline similarly to that described under “Data integration and the identification of NEUROG3+ cells”.

### Immunofluorescent Staining

Formalin-fixed, paraffin-embedded (FFPE) tissue sections as well as frozen tissue sections were obtained from the biobanks of the University of Pennsylvania and Network for Pancreatic Organ Donors with Diabetes (nPOD). Tissues from the following donors were used in the study: ICRH85 (18 day-old hispanic female), nPOD6407 (5 year-old Caucasian female), HPAP012 (18 year-old Caucasian female). The pancreatic tissue was harvested from deidentified cadaver donors and is not considered to be human subjects. The research is reviewed and exempted by the FSU IRB committee. FFPE sections were de-paraffinized with xylene and carried through sequential rehydration from 100% Ethanol to 70% Ethanol before being transferred to water. Heat-induced antigen retrieval was performed in a pressure cooker at 120°C for 20 min in the Tris-EDTA buffer (10mM Tris, 1mM EDTA, pH 9.0). Frozen sections were fixed with 4% PFA for 10 minutes at room temperature. The following primary antibodies were used: GHRL (Santa Cruz Biotechnology, sc-293422, clone 2F4, 1:500), NEUROG3 (R&D systems, AF3444, 1:300), Ki67 (Invitrogen, MA5-14520, clone SP6, 1: 250), Glucagon (Santa Cruz Biotechnology, sc-13091, 1:300), Somatostatin (Santa Cruz Biotechnology, sc-13099, 1:300), Insulin (Invitrogen, 701265, clone 19H4L12, 1:300). The following secondary antibodies were uses: Cy2-anti-mouse (Jackson ImmunoResearch, 715-225-150), Cy3-anti-rabbit (Jackson ImmunoResearch, 711-165-152), Cy5-anti-sheep (Jackson ImmunoResearch, 713-175-147). All secondary antibodies were applied at 1:300 dilution. Slide scanning images were taken with an Olympus microscope at 20x/0.75NA. Confocal images were captured with Leica SP7 at 63x/1.4NA.

### Image Analysis

To quantify the percentage of NEUROG3+ cells, an automated image analysis pipeline was applied on slide scans similar as described before (41). Briefly, DNA was used to identify nuclei (primary objects). The primary objects were expanded 10 pixels to demarcate cell boundaries. The intensities of GHRL or INS+GCG+SST channels were scaled to the range between 0-1. The intensities of these hormone channels were then measured in each cell. Endocrine cells were identified based on standard deviation of the staining intensities above a minimum value of 0.05. Outlines from endocrine cells identified using this pipeline were projected back onto the original endocrine staining images for visual inspection to confirm the appropriate cell type calling threshold. The pipeline was implemented in CellProfiler (3.1.8) (42). The NEUROG3+ cells among different populations were manually counted by two different investigators. The counting results from these two investigators showed good agreement.

## Results

### The Identification of NEUROG3+ Cells From Four Single-Cell RNA-Seq Datasets of Postnatal Human Islet Cells

**Figure 1:**
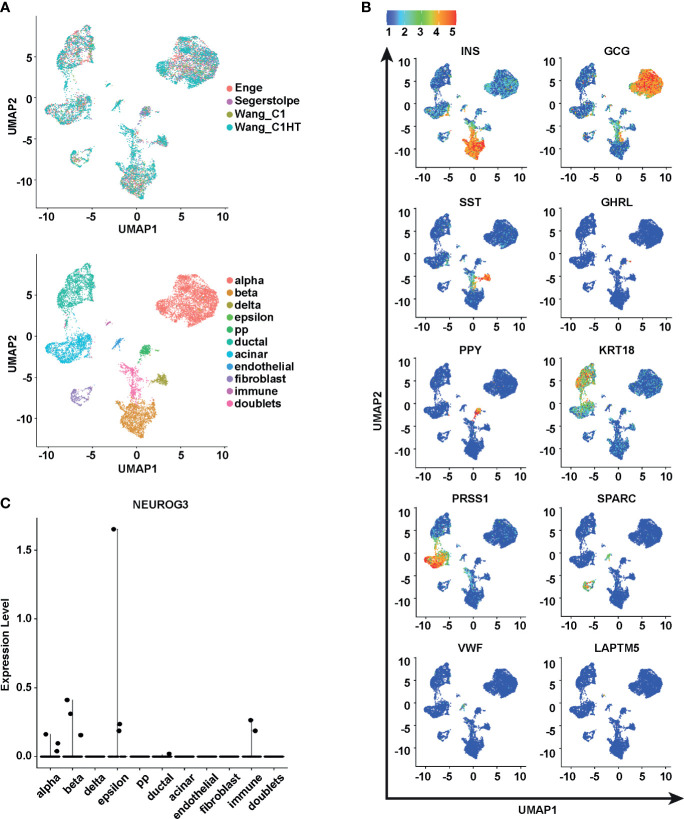
The identification of NEUROG3+ cells from 11,174 postnatal human pancreatic cells. **(A)** Uniform Manifold Approximation and Projections (UMAPs) are used to visualize the single-cell RNA-seq data post alignment. Top panel, cells are colored according to their study sources. Bottom panel, cells are colored according to cell types. **(B)** Relative expression of known pancreatic cell-type markers. The color scale is based on normalized expression values of transcripts in each UMAP panel. **(C)** Scatter plot exhibits the expression levels of NEUROG3 in different populations.

Expecting NEUROG3+ cells to be rare, we examined four large single-cell RNA-seq datasets, including two from our own research, designed to study postnatal human islet cells (27–31). Due to batch effects, single cells clustered by their sources rather than cell types, although donor mixing within each source appeared to be homogenous (**Supplementary Figure S1A**). Therefore, we harmonized the four datasets with the Seurat SCTransform pipeline (34). This strategy effectively controlled batch effects (**Figure 1A**). In the integrated data, cells clustered based on cell types, as illustrated by the expression patterns of marker genes (**Figure 1B**). We identified a total of ten cell clusters including all the major pancreatic cell types, together with one cluster of potential doublets displaying a mixture of endocrine markers (**Figure 1A** and **Table S1**). 11,174 cells passed quality control from the four datasets combined. Among them, 5,264 cells contained cell type labels from the original publications. Ninety-seven percent of our *de novo* cell-type annotation was in agreement with the original cell type labels, confirming the accuracy of our pipeline.

We examined the expression of NEUROG3 in the integrated data. We observed 12 cells with detectable NEUROG3 expression (**Figure 1C** and **Table S2**). To confirm the authenticity of these NEUROG3 transcripts, we directly visualized the aligned reads in the *NEUROG3* genomic region. In all the 12 cells, we observed clear indications of fragments aligned to NEUROG3 exons (**Supplementary Figure S2**). This result confirmed that these 12 cells had *bona fide* transcription from *NEUROG3*.

Assuming no bias in single-cell RNA-seq sample selection, the overall detection rate of NEUROG3+ cells was less than 0.1%. The 12 NEUROG3+ cells originated from multiple donors with various conditions (six control donors, one donor with type 1 diabetes, and three donors with type 2 diabetes), and of different ages (18 days to 57 years old), confirming that NEUROG3+ cells exist in postnatal human pancreata regardless of health status and age (**Table S2**). The identification of these cells in donors with diabetes is significant since it points to the possibility of utilizing these cells for diabetes treatment in an autologous manner.

These 12 cells were assigned as different cell types (three as alpha cells, three as beta cells, three as epsilon cells, one as a ductal cell, and two as immune cells). None of the NEUROG3+ cells had delta, pancreatic polypeptide, or acinar cell labels, probably due to the relatively low cell number in the single-cell RNA-seq data corresponding to each of these populations. In contrast, three NEUROG3+ cells were annotated as the epsilon cell type where a total of 35 epsilon cells were detected, given a percentage of 8.6%. The disproportionally high percentage of NEUROG3+ cells clustered together with epsilon cells implies a close transcriptional relationship between these two cell types. The expression of NEUROG3 in immune cells is curious since this has never been reported. Nonetheless, RNA-seq data from human tissues from the GTEx Portal (dbGaP accession number phs000424.vN.pN.) and cell lines from the Human Protein Atlas (43) both corroborated the finding and indicated the presence of NEUROG3 in immune cells (**Supplementary Figures S1B, C**). Due to limited knowledge, we did not include the two NEUROG3+ cells labeled as immune cells in our further analyses.

The rare but non-zero recoveries of NEUROG3+ cells in postnatal pancreata confirms their existence in the mature human pancreas. Moreover, the identification of these cells showcases the superior resolution and power of single-cell RNA-seq technology.

### Signatures of the NEUROG3+ Cells

We sought to identify genes differentially expressed in the NEUROG3+ cells. To mitigate the batch effects due to different data sources, cell origins as well as cell type labels were included as covariates in the linear model when we compared the gene expression profiles of the 10 NEUROG3+ cells with other pancreatic epithelial cells using limma (37). At a false discovery rate of 5%, we identified a total of 249 genes differentially expressed in the NEUROG3+ cells. Among these 249 genes, 247 were upregulated and 2 were downregulated in the NEUROG3+ cells compared with other cell types (**Table S3**).

**Figure 2:**
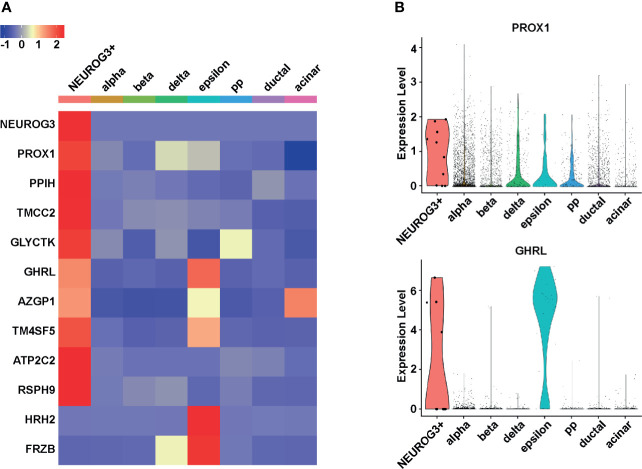
NEUROG3+ cells have unique gene expression signatures. **(A)** Heatmap demonstrates the average expression of the 10 most upregulated and two most downregulated transcripts in NEUROG3+ cells compared to other pancreatic epithelial cell types. The color scale is based on the normalized Z-score of each transcript. **(B)** Violin plots display the expression levels of PROX1 and GHRL in different cell populations. Each dot represents one cell.

As expected, after ranking these differentially expressed genes by fold change, the most highly enriched mRNA in the NEUROG3+ cells was NEUROG3 (**Figure 2A**). Following NEUROG3, top enriched transcripts in the NEUROG3+ cells included (**Figure 2A**): Prospero Homeobox 1 (PROX1); Peptidylprolyl Isomerase H (PPIH); Transmembrane And Coiled-Coil Domain Family 2 (TMCC2); Glycerate Kinase (GLYCTK); Ghrelin (GHRL); Alpha-2-Glycoprotein 1, Zinc-Binding (AZGP1); Transmembrane 4 L Six Family Member 5 (TM4SF5); ATPase Secretory Pathway Ca2+ Transporting 2 (ATP2C2); and Radial Spoke Head Component 9 (RSPH9).

Among the genes that were upregulated in the NEUROG3+ cells, PROX1 (**Figure 2B**, upper panel) and GHRL (**Figure 2B**, lower panel) have known functions in the pancreas. PROX1 encodes a homeobox protein; it is involved in pancreas organogenesis, endocrine fate specification, and beta cell maturation (44, 45). GHRL is considered an epsilon cell marker as it encodes a hormone that is uniquely secreted by endocrine epsilon cells.

Other upregulated genes are not well studied in the context of pancreatic endocrine development. PPIH is a component of the pre-mRNA processing complex and mediates pre-mRNA splicing (46). TMCC2 interacts with amyloid precursor protein and may play a role in neurodegeneration (47). GLYCTK is involved in serine and fructose metabolism (48). AZGP1 stimulates lipolysis and is involved in the maintenance of epithelial identity (49, 50). TM4SF5 is a member of the tetraspanin family and functions in cell cycle progression, cell migration, and epithelial-mesenchymal transition (51). ATP2C2 is involved in ATP-dependent calcium transport (52). RSPH9 is a component of the radial spokes within cilia, sperm, and flagella (53). Four out of the 10 most upregulated transcripts, including TMCC2, AZGP1, TM4SF5, and ATP2C2, encode putative transmembrane proteins. These proteins can be potentially exploited for cell surface labeling followed by flow cytometry-based enrichment of live NEUROG3+ cells.

The two transcripts that were significantly downregulated in the NEUROG3+ cells were Histamine Receptor H2 (HRH2) and Secreted Frizzled Related Protein (FRZB) (**Figure 2A**). HRH2 mediates histamine signaling and regulates cell proliferation (54). FRZB inhibits Wnt signaling (55). Several studies have shown that FRZB is downregulated in pancreatic cancer, suggesting that this protein may play a role in pancreatic cell fate maintenance (56).

**Figure 3:**
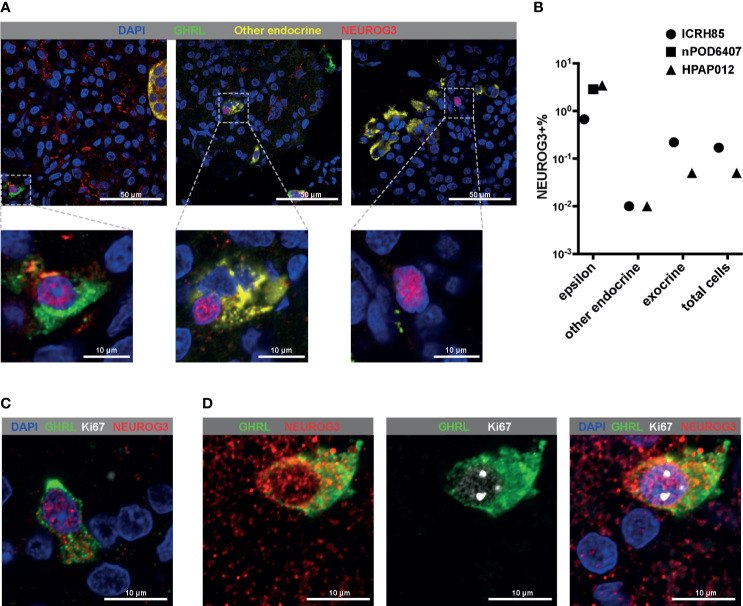
Immunofluorescent staining confirms the high frequency of NEUROG3 expression within epsilon cells. **(A, C, D)** All images shown are a single confocal Z-section. **(A)** Representative images showing the positive nuclear labeling of NEUROG3 in epsilon cells (left panel), other endocrine cells (middle panel) and exocrine cells (right panel) in postnatal human pancreatic tissue sections. Zoomed-in views of boxed regions are shown at the bottom of each panel. Color channels are: DAPI, blue; GHRL, green; INS+GCG+SST (a combination of three antibodies against individual proteins), yellow; NEUROG3, red. **(B)** Quantifications of NEUROG3+ cells in different populations. All cells from each section of the donor tissues were counted. Each data point represents aggregated results from one donor. “Other endocrine” corresponds to beta (INS+) or alpha (GCG+) or delta (SST+) cells. **(C, D)** Co-labeling of GHRL, NEUROG3 and Ki67. **(C)** The majority of NEUROG3+ cells are not proliferating. **(D)** in rare instances, coexpression of Ki67 and NEUROG3 can be observed. Color channels are: DAPI, blue; GHRL, green; Ki67, white; NEUROG3, red.

The upregulation of GHRL in the NEUROG3+ cells was consistent with our observation that there was an enrichment of NEUROG3+ cells in the epsilon population. To confirm the results from single-cell RNA-seq, we co-stained GHRL and NEUROG3 proteins alongside other hormone makers in postnatal human pancreatic tissue sections (**Figure 3A**). The immunostaining pattern corroborated with single-cell RNA-seq findings: we noted instances of NEUROG3 antibody positive labeling in all tissue sections examined. Nuclear NEUROG3 staining could be located in multiple cell types including epsilon cells, non-epsilon endocrine cells, as well as pancreatic exocrine cells (**Figure 3A**). The percentage of NEUROG3+ cells varied among different donors, but were consistently higher in the epsilon population (0.67-3.42%) compared with the frequencies of NEUROG3+ cells in other endocrine (0.01%) and exocrine cells (0.05-0.22%) (**Figure 3B** and **Table S4**). To be noted, one of the pancreatic tissues was from an 18 year-old young adult. The identification of NEUROG3+ cells at the protein level in this donor provided further evidence of the existence of the NEUROG3+ cells in mature pancreata. To further examine the regenerative potential of the NEUROG3+ cells *in vivo*, we co-labelled the pancreatic tissue with NEUROG3 and the proliferation marker Ki67. The majority of NEUROG+ cells were post-mitotic (**Figure 3C**). However, in rare cases, we observed co-expression of NEUROG3 and Ki67 (**Figure 3D**). This result indicates that at least a fraction of human postnatal NEUROG3+ cells could proliferate.

### The Gene Regulatory Network of NEUROG3

**Figure 4:**
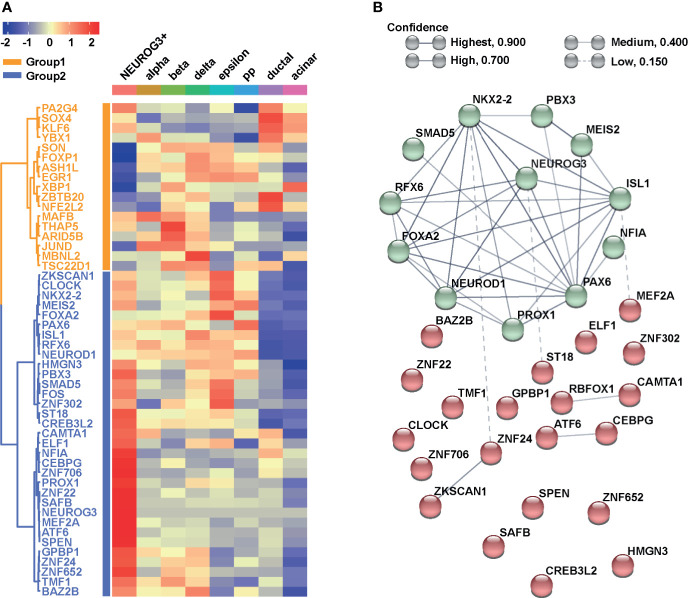
NEUROG3+ cells contain an organized transcriptional network. **(A)** Heatmap presents the average expressions of the top 50 transcription factors (TFs) expressed in NEUROG3+ cells compared with other cell types, along with hierarchical clustering. **(B)** STRING analysis displays connectivity of Group 2 TFs. Nodes are colored based on their k-mean cluster memberships. The thickness of connection lines is reflective of the strength of data supporting the interactions.

To investigate the regulatory framework of NEUROG3, we next focused on transcription factors (TFs). We ranked all the TFs based on their average expressions in the NEUROG3+ cells and focused on the top 50 most highly expressed. We examined the levels of the 50 TFs in the NEUROG3+ cells relative to other pancreatic cell types. Hierarchical clustering based on Pearson correlation revealed that the 50 TFs fell into two major groups, with genes in Group 2 generally displaying higher expression in the NEUROG3+ cells compared to other cells (**Figure 4A**). We proceeded to perform network analysis with STRING using all the TFs in Group 2 (38). We found that the Group 2 TFs can be further separated into two clusters: one cluster of TFs highly connected to each other and to NEUROG3 (NEUROG3 hub) and the other cluster of TFs loosely connected to each other and to the NEUROG3 hub (**Figure 4B**).

Within the NEUROG3 hub, some TFs have been reported in mice to function in endocrine fate specification (**Figure 4B**). Their functions are likely to be conserved here. For example, we recognized TFs that are considered to be the direct targets of NEUROG3. These include ISL LIM Homeobox 1 (ISL1) (1) Neuronal Differentiation 1 (NEUROD1) (57), NK2 Homeobox 2 (NKX2-2) (58), Paired Box 6 (PAX6) (59), and Regulatory Factor X6 (RFX6) (60). We also identified an upstream regulator of NEUROG3: Forkhead Box A2 (FOXA2) (61). It was reported that NEUROG3 and FOXA2 are co-expressed and functionally synergize during endocrine differentiation (61).

Within the NEUROG3 hub, other TFs were previously uncharacterized regarding their functional interactions with NEUROG3 (**Figure 4B**). These included Meis Homeobox 2 (MEIS2), PBX Homeobox 3 (PBX3), PROX1, Nuclear Factor IA (NFIA), and SMAD Family Member 5 (SMAD5). MEIS2 and PBX3 are homeobox proteins. They were shown to form a complex that targets PAX6 during pancreatic development and endocrine specification (62). Further, MEIS2 interacts with PBX1b (a paralog of PBX3) and PDX1 and activates pancreatic acinar cell programs (63). As described above, PROX1 plays a role in pancreatic development and endocrine differentiation. NFIA plays a role in stem cell maintenance and cellular differentiation (64). NFIA promotes pancreatic endocrine differentiation through Notch signaling regulation (65). Finally, SMAD5 is a component of the TGFβ signaling pathway. To begin to understand the pancreatic-specific molecular program involving these TFs, we accessed the Islet Regulome Browser to visualize the local genomic profiles surrounding each TF (66). Intriguingly, promoter and enhancer regions of all these five TFs are bound by essential factors involved in pancreatic organogenesis in the pancreatic progenitor cells (**Supplementary Figure S3**). This result indicates that these TFs may cooperate with NEUROG3 and play a role in the endocrine differentiation process in humans.

### Comparison of Postnatal NEUROG3+ Cells With NEUROG3+ Cells in Human Fetal Pancreata

**Figure 5:**
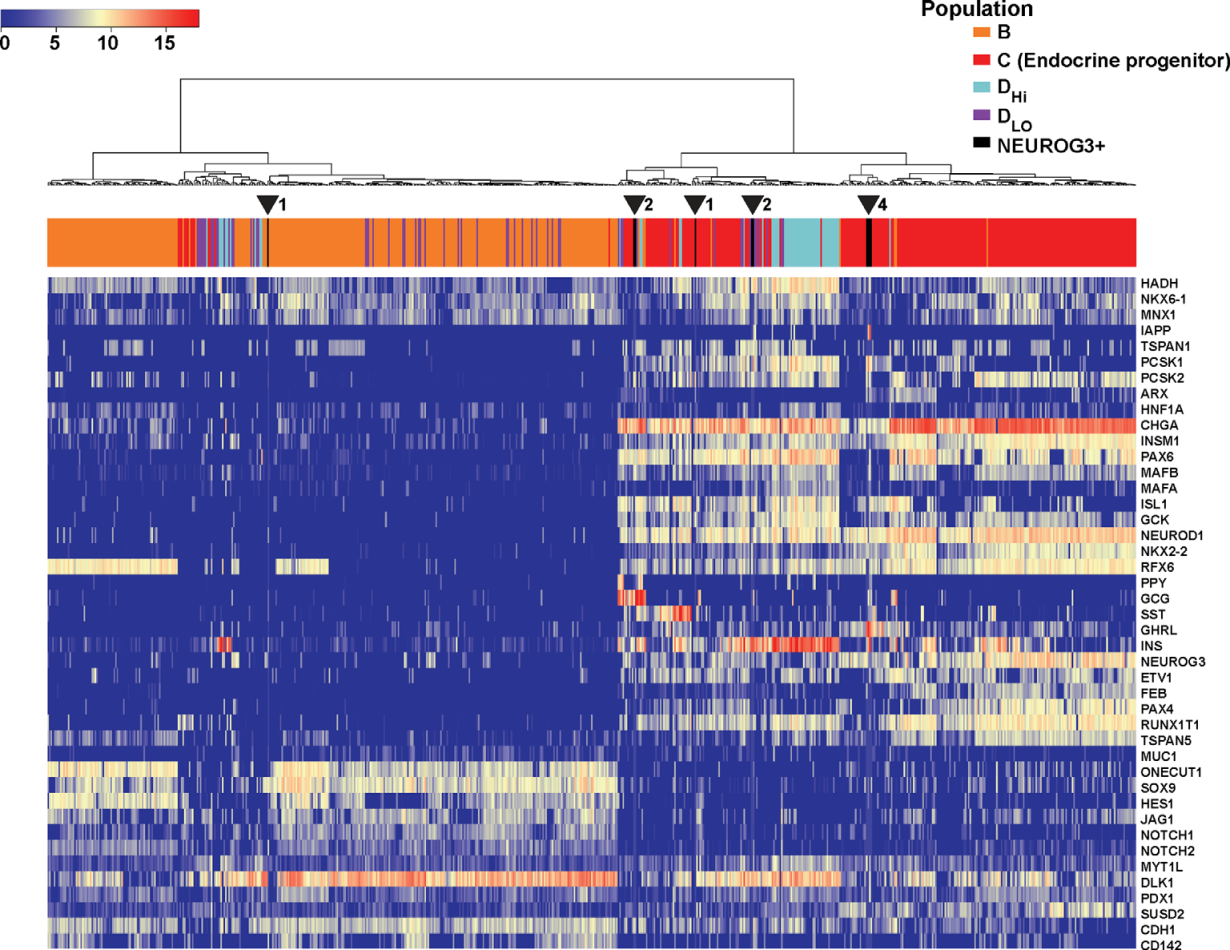
NEUROG3+ cells in the postnatal human pancreas cluster with NEUROG3+ cells from the fetal pancreas. Heatmap displays the relative gene expression of postnatal NEUROG3+ cells and single cells isolated from the human fetal pancreas, along with hierarchical clustering. Dendrograph displays color-coded populations: B (orange), C (red), D_Hi_ (light blue), D_lo_ (violet) and NEUROG3+ cells (black). Population labels are consistent with the original publication (39). Population C is the endocrine progenitor population (EP). Nine out of 10 NEUROG3+ cells cluster with EP. The location of NEUROG3+ cells are indicated by arrowheads, with the number of NEUROG3+ cells in each location denoted next to each arrowhead.

We examined whether NEUROG3+ cells in the postnatal human pancreas shared characteristics with NEUROG3+ cells during development. Available data are in the form of single-cell qPCRs consisting of 683 single cells isolated from human fetal pancreata at 9 weeks of development (39). We extracted the gene expression values of the 10 NEUROG3+ cells and combined them with the qPCR data from human fetal pancreatic cells. We applied hierarchical clustering based on the Pearson correlation distance between different cells with Ward’s linkage. This algorithm placed 9 out of the 10 NEUROG3+ cells together with the endocrine progenitor population in the human fetal dataset [Endocrine progenitors labeled as Population C in the original publication (39); **Figure 5**]. The resemblance in gene expression of NEUROG3+ cells from the postnatal human pancreas with NEUROG3+ cells during development suggests that these postnatal NEUROG3+ cells are likely resident endocrine progenitors.

### Comparison of Postnatal NEUROG3+ Cells With NEUROG3+ Cells Derived *In Vitro*

The 10 NEUROG3+ cells identified in our study did not form a unique cluster when visualized with UMAP. We reasoned that by increasing the NEUROG3+ cell numbers, clusters and even substructures of NEUROG3+ cells might emerge. Towards this goal, we explored seven additional single-cell RNA-seq datasets on human pancreatic cells with more than 36,000 aggregated single cells (67–73). Surprisingly, no more NEUROG3+ cells were identified from the seven datasets. To be noted, the four datasets containing NEUROG3+ cells in our study all utilized platforms/chemistries that have the highest sensitivities and lowest transcripts dropouts (Smart-seq2 method and Fluidigm C1 platforms) (30). This suggests that the lack of NEUROG3+ cells in the other studies may be due to the detection limit of single-cell RNA-seq chemistry.

Contrary to the limited data on NEUROG3+ cells from human pancreata, several large single-cell RNA-seq datasets containing NEUROG3+ cells derived *in vitro* were recently published. We selected one dataset where NEUROG3+ cells were isolated from early stage 6 of differentiation from human embryonic stem cells to beta cells (40). We aligned the *in vitro* dataset with our integrated single-cell RNA-seq dataset (**Supplementary Figures S4A, B**). The endocrine and ductal cells derived *in vitro* were mapped onto various mature endocrine or ductal cell regions, consistent with what was described by Krentz et al. (**Supplementary Figure S4C**; see also Figure 6A from Krentz et al.) (40). Under this alignment, the NEUROG3+ cells derived *in vitro* and the 10 NEUROG3+ cells we have identified diffused into multiple regions of the mature pancreatic cell types rather than cluster together (**Supplementary Figure S4D**). This observation suggests that there might be transcriptional differences between endogenous NEUROG3+ cells and NEUROG3+ derived *in vitro*. Nevertheless, we could conclude that, similar to the 10 naturally occurring NEUROG3+ cells we have analyzed, the NEUROG3+ derived *in vitro* were highly heterogeneous. We could further infer that different cell fates might have been established before or with the onset of NEUROG3 expression. Recent study indicated that cell fates were regulated *via* DNA methylation shifts parallel to the initiation of NEUROG3 expression (11).

### Validation of Single-Cell RNA-Seq Results With an Independent Single-Nucleus RNA-Seq Dataset From Neonatal Human Pancreas

During the preparation of the manuscript, a human pancreas atlas containing single-nucleus RNA-seq data from neonatal human pancreas became available (74). Because of the differences of this atlas dataset compared to the single-cell RNA-seq datasets we explored with respect to starting materials (snap frozen pancreata versus isolated human islets), sample types (single-nucleus versus single-cell), and sequencing depth (median reads 775 reads/nucleus versus >300,000 reads/cell), we did not opt to integrate this atlas dataset into our analysis. Rather, we considered this dataset as an independent validation. In this atlas dataset, 55 NEUROG3+ cells were observed. Similar to the single-cell RNA-seq datasets, the NEUROG3+ cells from the atlas dataset were distributed among multiple pancreatic cell types (**Supplementary Figure S5A** and **Table S5**). The frequency of NEUROG3+ cells was again highest in the epsilon cell population (7%). Furthermore, among the top 50 most highly expressed transcription factors in both of the single-cell and single-nucleus datasets, 16 of them were shared between the two datasets (**Supplementary Figure S5B**). Due to the technical difference in mRNA populations captured from the single-nucleus RNA-seq data compared with single-cell RNA-seq data, a direct comparison of differentially expressed genes in the NEUROG3+ cells could not be performed. However, the significant overlap of transcription factor expressions as well as the enriched epsilon cell type in the NEUROG3+ population confirmed our single-cell analysis and indicated that human NEUROG3+ harbors a stable set of molecular signatures. Further, since the single-nucleus RNA-seq data were enriched with nascent transcripts, the shared transcriptional signatures between the NEUROG3+ nuclei and the NEUROG3+ cells indicate that the 10 NEUROG3+ cells we identified were likely to have active NEUROG3 transcription.

## Discussion

Utilizing existing single-cell RNA-seq datasets, we performed the first systematic characterization of the transcriptomics of NEUROG3+ cells from humans. Our study confirmed the presence of NEUROG3+ in the postnatal human pancreas. We provided a unique set of markers that are enriched in these NEUROG3+ cells. We described a transcriptional network involving *NEUROG3*. This network contained known as well as previously uncharacterized regulators for endocrine differentiation. We validated the RNA-seq result and demonstrated the co-expression of NEUROG3 with mature hormone markers at the protein level. We further confirmed the high percentage of NEUROG3+ cells in the epsilon population. Comparisons of postnatal human NEUROG3+ cells with NEUROG3+ cells during development revealed conserved molecular programs. Despite the small number of NEUROG3+ cells detected and analyzed in our integrated single-cell RNA-seq dataset, our results were meaningful and could be reproduced with an independent single-nucleus RNA-seq dataset. We conclude that postnatal NEUROG3+ cells are likely resident endocrine progenitors that can be further functionally and bioinformatics explored to gain insights on diabetes therapies.

We characterized a transcriptional network in the human NEUROG3+ cells and identified known as well as novel candidates that functionally interact with NEUROG3. Several transcription factors, including FOXA2, ISL1, NEUROD1, NKX2-2, PAX6, and RFX6 are recognized as canonical targets of NEUROG3. In contrast, other transcription factors, including MEIS2, NFIA, PBX3, PROX1, and SMAD5, are not well characterized in the context of NEUROG3. Their high expression in the NEUROG3+ cells, their high connectivity with the NEUROG3 hub, and the co-occupancy of pancreatic master regulators in the enhancers of these genes indicate that these genes may play a role in the specification of pancreatic endocrine cell fate.

One unique feature of human NEUROG3+ cells is the predominant classification of NEUROG3+ cells as epsilon cells – an endocrine population generally considered to be terminally differentiated. This finding is supported by the co-expressing of NEUROG3 with epsilon cell marker GHRL at both the RNA and the protein level. The upregulation of GHRL in the postnatal NEUROG3+ cells coincides with a recent report showing the overlapping expression of GHRL and NEUROG3 in the human fetal pancreas (39). This result indicates that GHRL is a potential marker for NEUROG3+ endocrine progenitor cells. Our finding possibly posits epsilon cells earlier in the pancreatic endocrine differentiation cascade and implies a progenitor-like state of epsilon cells. This postulation is in line with a previous lineage tracing study demonstrating that at least a proportion of epsilon cells were multipotent and could give rise to other endocrine cell types (75). As an additional piece of evidence supporting the similarity of NEUROG3+ cells and epsilon cells, trajectory reconstruction of pancreatic endocrine differentiation with single-cell RNA-seq data of mouse embryonic pancreas placed epsilon cells interspersed with endocrine progenitors (76). In the process of quantifying the immunostaining of the pancreatic tissue sections for this paper, we occasionally noticed Ki67 positive signals in epsilon cell clusters. This observation strongly suggests that human epsilon cells can self-renew. Whether epsilon cells in humans display multipotent differentiation potential awaits further studies that creatively employ labeling and lineage tracing strategy.

Both the NEUROG3+ cells from postnatal human pancreata and the NEUROG3+ derived *in vitro* scattered into multiple regions of mature pancreatic cell types. These results suggest that human NEUROG3+ cells are heterogeneous and have been lineage primed to specific cell fates. In the mice, it is shown that NEUROG3+ cells consist of two populations with either low or high NEUROG3 expression. These two populations have distinct cell-cycle and differentiation properties, with NEUROG3-low cells more proliferative and multipotent, while NEUROG3-high cells less proliferative and unipotent (77–79). Due to the limited number of human NEUROG3+ cells identified, we could not categorize the NEUROG3 expression levels and further resolve the temporal orders of these NEUROG3+ cells to infer the differentiation trajectory. It is thus unclear whether the mouse equivalent NEUROG3 subpopulations exist in humans. From immunostaining, we did observe instances where NEUROG3+ cells were co-labelled with the proliferation marker Ki67, suggesting that at least a fraction of human postnatal NEUROG3+ cells could proliferate.

In summary, our study is the first systematic characterization of the gene expression landscape of NEUROG3+ cells from the postnatal human pancreas. This work provides a resource for the biomedical research community to further explore in order to gain more insights on NEUROG3+ cells in humans. Because diabetes is mostly diagnosed in adults, understanding the signatures of NEUROG3+ cells in the post-developmental human pancreas is important from the point of diabetes treatment. However, critical gaps still exist before we can establish the therapeutic values of the NEUROG3+ cells. For example, it is unclear whether the NEUROG3+ cells play a significant role in the pancreatic cell turnover during normal homeostasis and in pathological conditions. Neither do we know the differentiation cascade from NEUROG3+ cells to mature endocrine cells. With the advance of massively parallel deep profiling single-cell RNA-seq technology and continued improvement in algorithms for data harmonization, further studies can help to fine map the NEUROG3+ cells. Moreover, experimental perturbation is required to enhance and complement the *in silico* findings to delineate the regulatory process of NEUROG3.

## Data Availability Statement

The datasets presented in this study can be found in online repositories. The names of the repository/repositories and accession number(s) can be found below:

https://www.ncbi.nlm.nih.gov/geo/, GSE81547, GSE83139, GSE154126
https://www.ebi.ac.uk/arrayexpress/, E-MTAB-5061
https://hpap.pmacs.upenn.edu, NA
https://ega-archive.org/studies/EGAS00001004653, EGAS00001004653.

## Author Contributions

HY made substantial contributions to the design, analysis, and interpretation of data. GX, CL, WW, AN, and JI made substantial contributions to the acquisition of data. YW made substantial contributions to conception and design, acquisition of data, analysis, and interpretation of data. YW is the guarantor of this work and, as such, has full access to all the data in the study and takes responsibility for the integrity of the data and the accuracy of the data analysis. All authors contributed to the article and approved the submitted version.

## Funding

Supports for this work are provided by the Integrated Islet Distribution – Islet Award Initiative (IIDP-IAI; (IIDP-IAI; https://iidp.coh.org/Investigators/Islet-Award-Initiative) and the Helmsley Charitable Trust George S. Eisenbarth nPOD Award for Team Science. This work is also supported by Florida State University start-up fund.

## Conflict of Interest

The authors declare that the research was conducted in the absence of any commercial or financial relationships that could be construed as a potential conflict of interest.

## Publisher’s Note

All claims expressed in this article are solely those of the authors and do not necessarily represent those of their affiliated organizations, or those of the publisher, the editors and the reviewers. Any product that may be evaluated in this article, or claim that may be made by its manufacturer, is not guaranteed or endorsed by the publisher.

## Supplementary Material

Supplementary Figure 1 The identification and characterization of NEUROG3+ cells from single-cell RNA-seq datasets. **(A)** UMAPs are used to visualize the single-cell RNA-seq data prior to alignment. Left panel, cells are colored according to their study sources. Right panel, cells are colored according to donor origins. Different datasets display large batch effects; while within each dataset, cells from different donors appear to be well mixed. **(B)** Relative NEUROG3 expressions in different human tissues. There are high levels of NEUROG3 in EBV-transformed lymphocytes (highlighted). NEUROG3 expression was accessed through the GTEx Portal on 08/04/2020 and dbGaP accession number phs000424.vN.pN. **(C)** Relative NEUROG3 expressions in different human cell lines. There are high levels of NEUROG3 in THP-1 and HMC-1 cell lines, both of myeloid origin. NEUROG3 expression was accessed through Human Protein Atlas V 19.3 on 08/04/2020.

Supplementary Figure 2 Genome browser view of the aligned reads in the 12 NEUROG3+ cells at the NEUROG3 genomic region. **(A)** In each of the 12 NEUROG3+ cells, there are fragments mapped to the exons of *NEUROG3*. **(B)** The boxed region in A is zoomed to single nucleotide resolution. Red dash line indicates the starting site of an adjacent transcript. The aligned fragments all have nucleotides mapped beyond the starting site of the adjacent transcript, confirming that these reads originate from NERUGO3 mRNA.

Supplementary Figure 3 Islet regulome snapshots show transcription factor binding sites in the promoter/enhancer regions of 5 novel regulators including **(A)** MEIS2, **(B)** NFIA, **(C)** PBX3, **(D)** PROX1, and **(E)** SMAD5.

Supplementary Figure 4 NEUROG3+ cells in the postnatal human pancreas do not cluster together with NEUROG3+ cells derived *in vitro*. **(A)** UMAP displays the effect of data alignment. Cells are colored according to their study sources. Krentz dataset contains NEUROG3+ cells derived from stage 6 day 1 of *in vitro* differentiation from human embryonic stem cells to beta cells. **(B)** UMAPs display relative marker gene expressions. Color scale is based on normalized expression values. **(C)** UMAP demonstrates the partial alignment of the Endo1, Endo2, Endo3, and Ductal cells from the Krentz data with mature beta, alpha, delta, and ductal cells correspondingly. **(D)** NEUROG3+ cells scatter into multiple regions of the UMAP rather than form a unique cluster. Left panel, the 10 NEUROG3+ cells in the current study are shown in red and the NEUROG3+ from the Krentz dataset are shown in yellow. Other cells are colored grey. EP, annotated NEUROG3+ endocrine progenitors. Right panel, the level of the NEUROG3 expression is shown.

Supplementary Figure 5 Validation of the integrated single-cell RNA-seq dataset with a single-nucleus RNA-seq dataset. **(A)** Scatter plot exhibits the expression levels of NEUROG3 in different populations. **(B)** Venn diagram demonstrates the overlap of the top 50 most highly expressed transcription factors in the datasets. The significance of the overlap is calculated based on hypergeometric distribution.
