# Supplementary figures and images for: Gene Signatures of NEUROGENIN3+ Endocrine Progenitor Cells in the Human Pancreas
Source: Front Endocrinol (Lausanne). 2021 Sep 8;12:736286. doi: 10.3389/fendo.2021.736286 (PMC8456125; doi:10.3389/fendo.2021.736286)

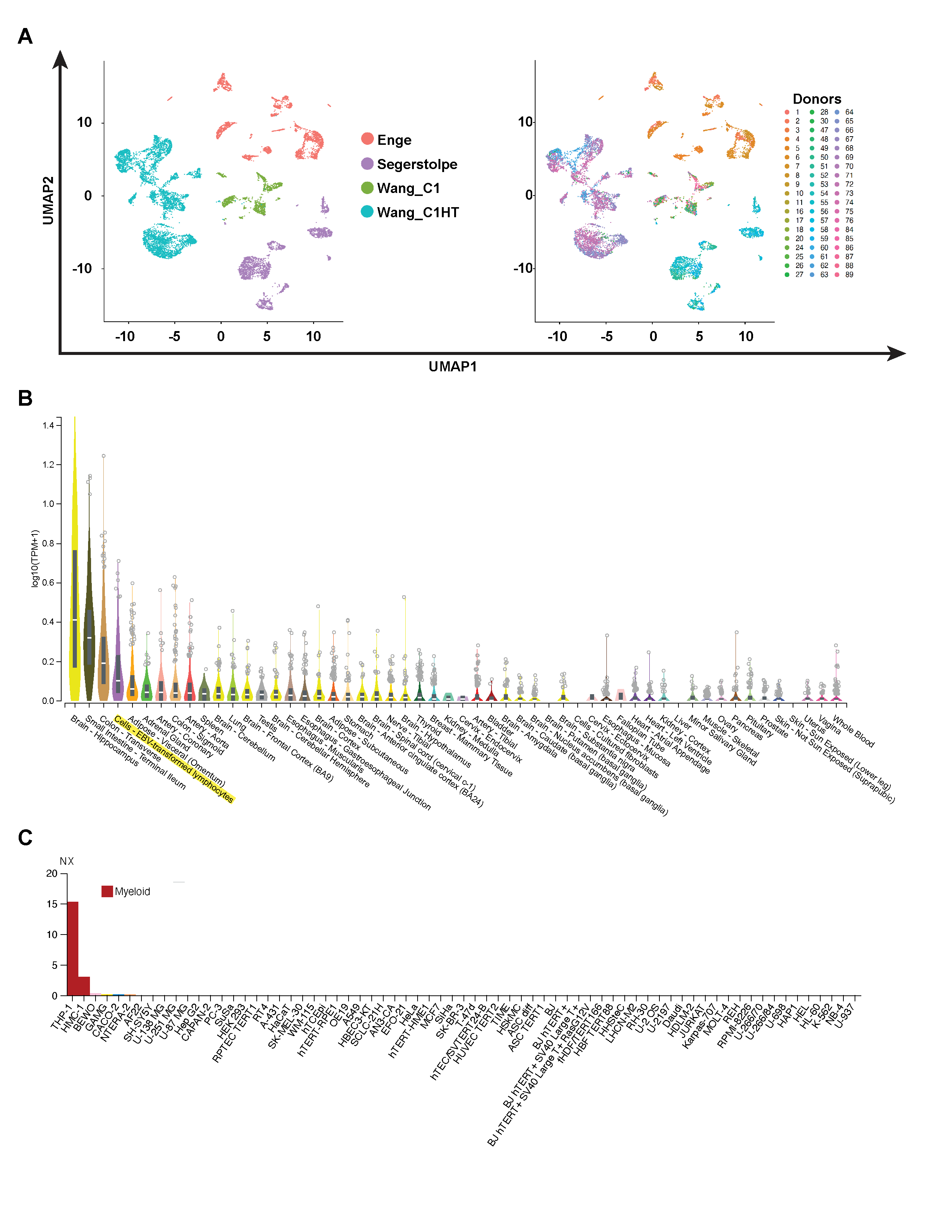

Supplement: Supplementary Figure 1 — The identification and characterization of NEUROG3+ cells from single-cell RNA-seq datasets. (A) UMAPs are used to visualize the single-cell RNA-seq data prior to alignment. Left panel, cells are colored according to their study sources. Right panel, cells are colored according to donor origins. Different datasets display large batch effects; while within each dataset, cells from different donors appear to be well mixed. (B) Relative NEUROG3 expressions in different human tissues. There are high levels of NEUROG3 in EBV-transformed lymphocytes (highlighted). NEUROG3 expression was accessed through the GTEx Portal on 08/04/2020 and dbGaP accession number phs000424.vN.pN. (C) Relative NEUROG3 expressions in different human cell lines. There are high levels of NEUROG3 in THP-1 and HMC-1 cell lines, both of myeloid origin. NEUROG3 expression was accessed through Human Protein Atlas V 19.3 on 08/04/2020. [file Image_1.tif]

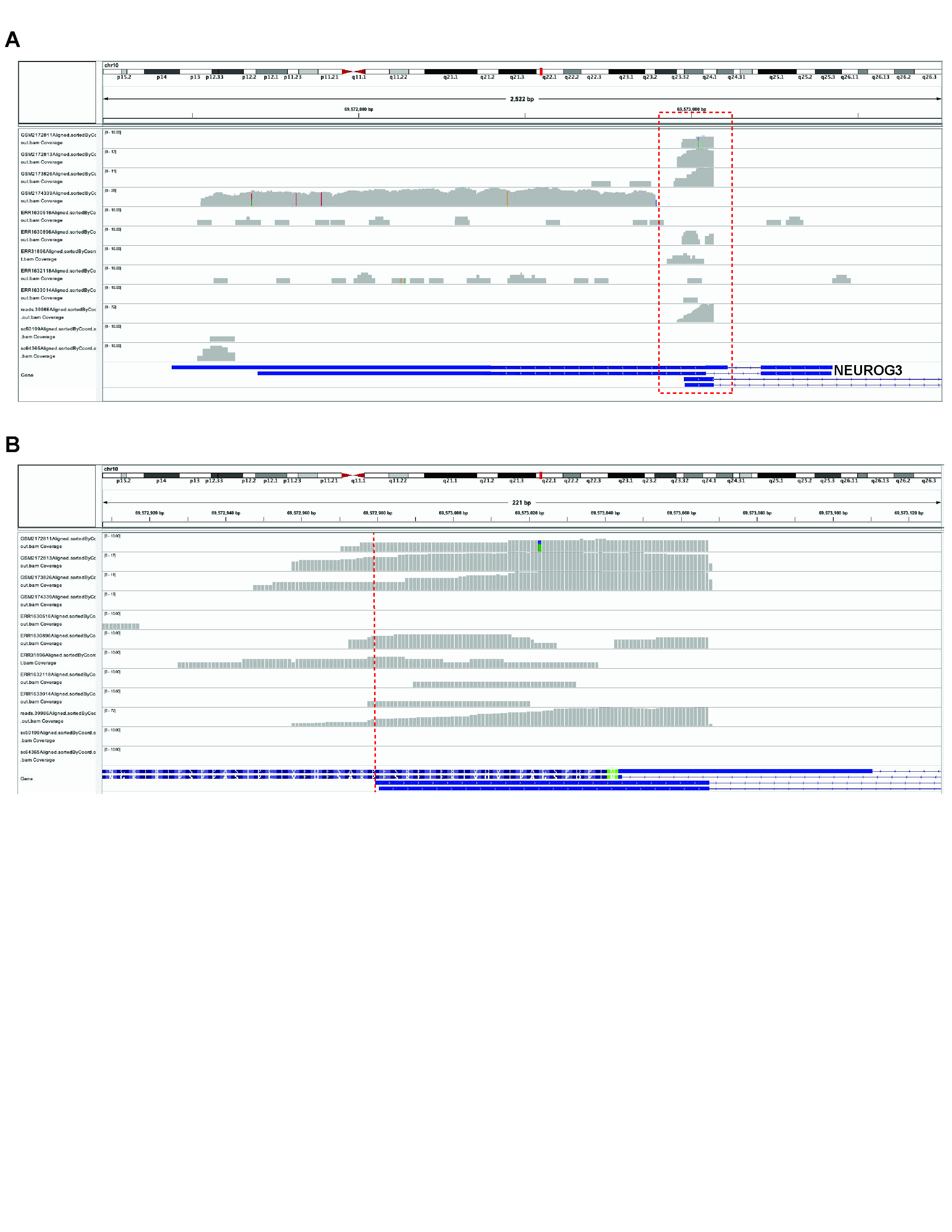

Supplement: Supplementary Figure 2 — Genome browser view of the aligned reads in the 12 NEUROG3+ cells at the NEUROG3 genomic region. (A) In each of the 12 NEUROG3+ cells, there are fragments mapped to the exons of NEUROG3. (B) The boxed region in A is zoomed to single nucleotide resolution. Red dash line indicates the starting site of an adjacent transcript. The aligned fragments all have nucleotides mapped beyond the starting site of the adjacent transcript, confirming that these reads originate from NERUGO3 mRNA. [file Image_2.tif]

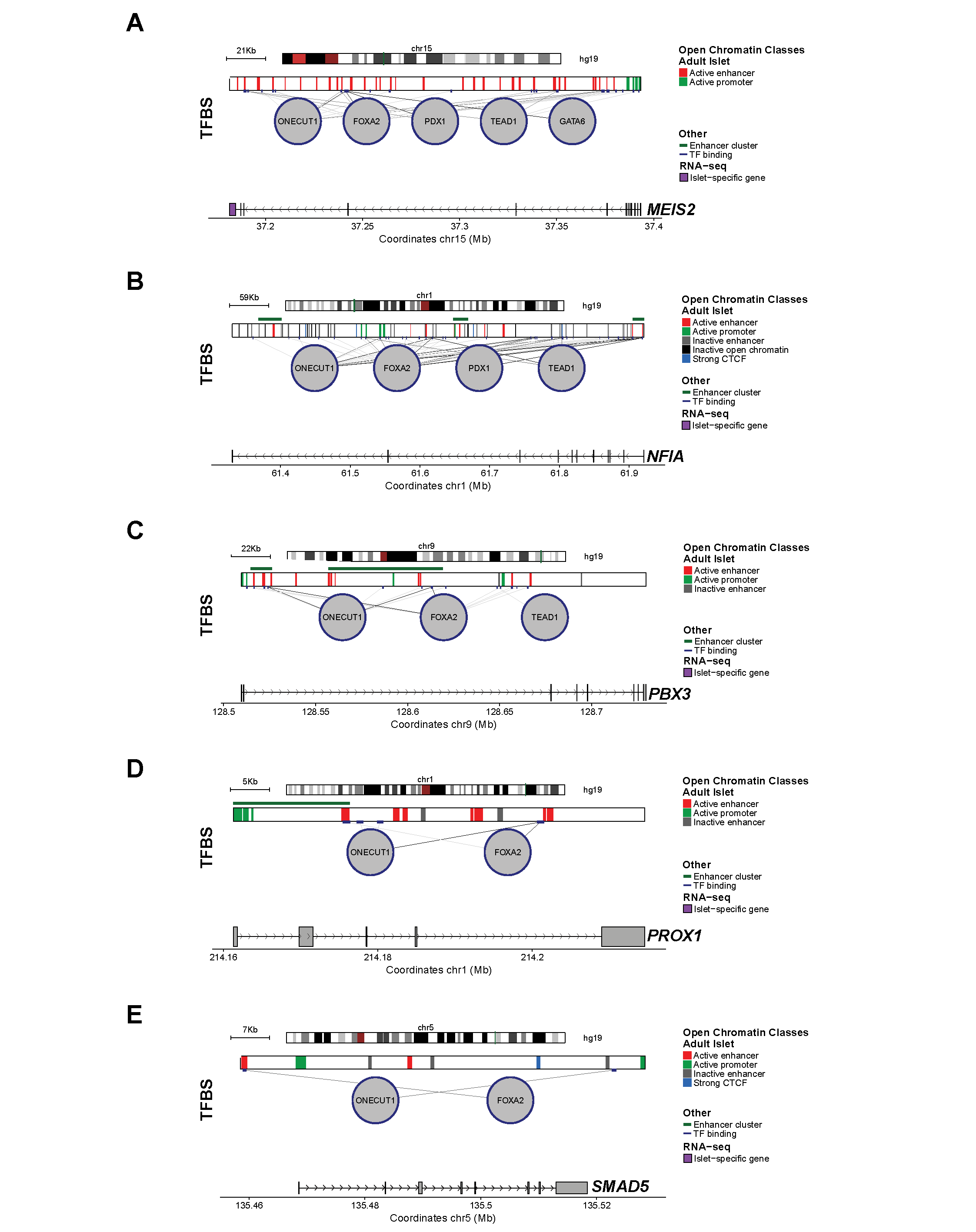

Supplement: Supplementary Figure 3 — Islet regulome snapshots show transcription factor binding sites in the promoter/enhancer regions of 5 novel regulators including (A) MEIS2, (B) NFIA, (C) PBX3, (D) PROX1, and (E) SMAD5. [file Image_3.tif]

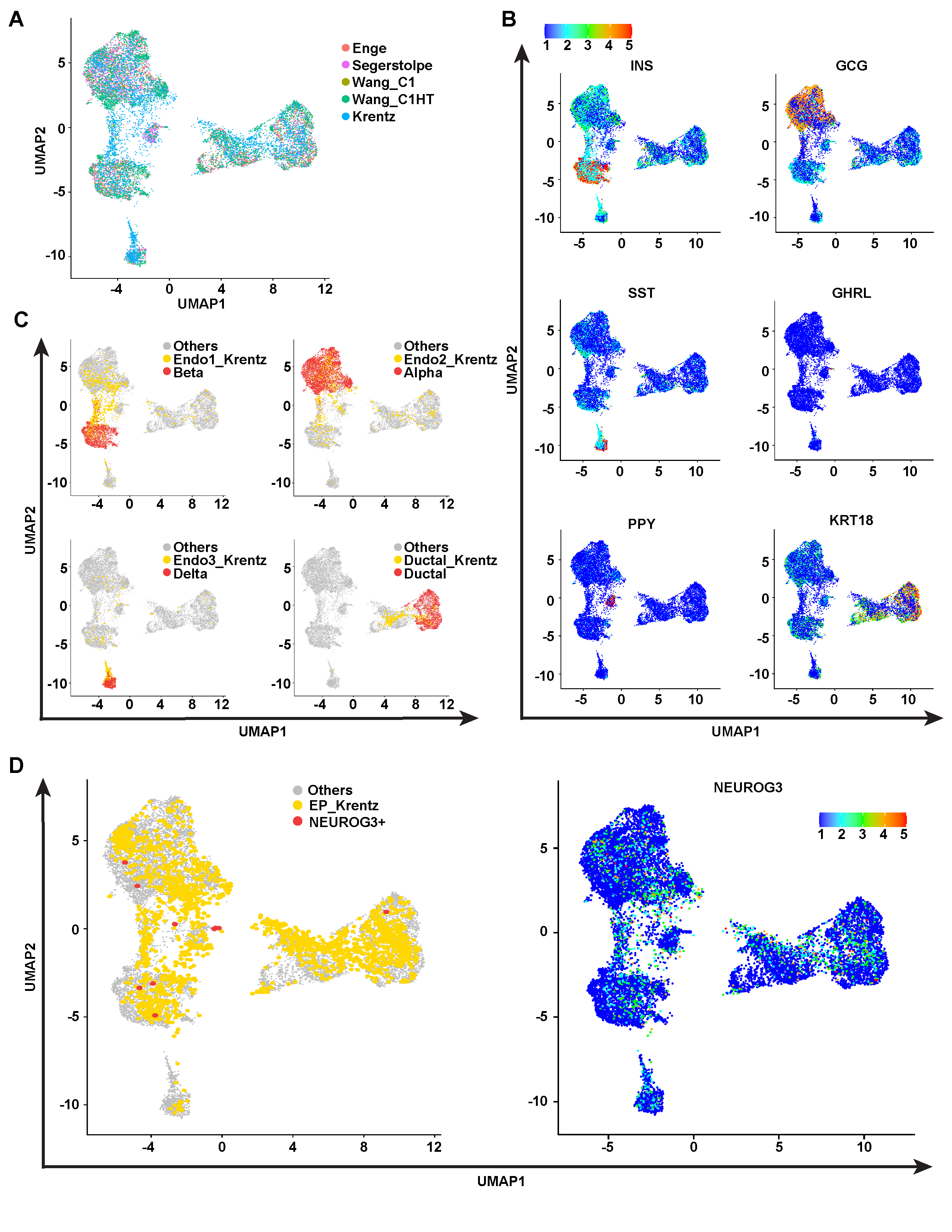

Supplement: Supplementary Figure 4 — NEUROG3+ cells in the postnatal human pancreas do not cluster together with NEUROG3+ cells derived in vitro. (A) UMAP displays the effect of data alignment. Cells are colored according to their study sources. Krentz dataset contains NEUROG3+ cells derived from stage 6 day 1 of in vitro differentiation from human embryonic stem cells to beta cells. (B) UMAPs display relative marker gene expressions. Color scale is based on normalized expression values. (C) UMAP demonstrates the partial alignment of the Endo1, Endo2, Endo3, and Ductal cells from the Krentz data with mature beta, alpha, delta, and ductal cells correspondingly. (D) NEUROG3+ cells scatter into multiple regions of the UMAP rather than form a unique cluster. Left panel, the 10 NEUROG3+ cells in the current study are shown in red and the NEUROG3+ from the Krentz dataset are shown in yellow. Other cells are colored grey. EP, annotated NEUROG3+ endocrine progenitors. Right panel, the level of the NEUROG3 expression is shown. [file Image_4.tif]

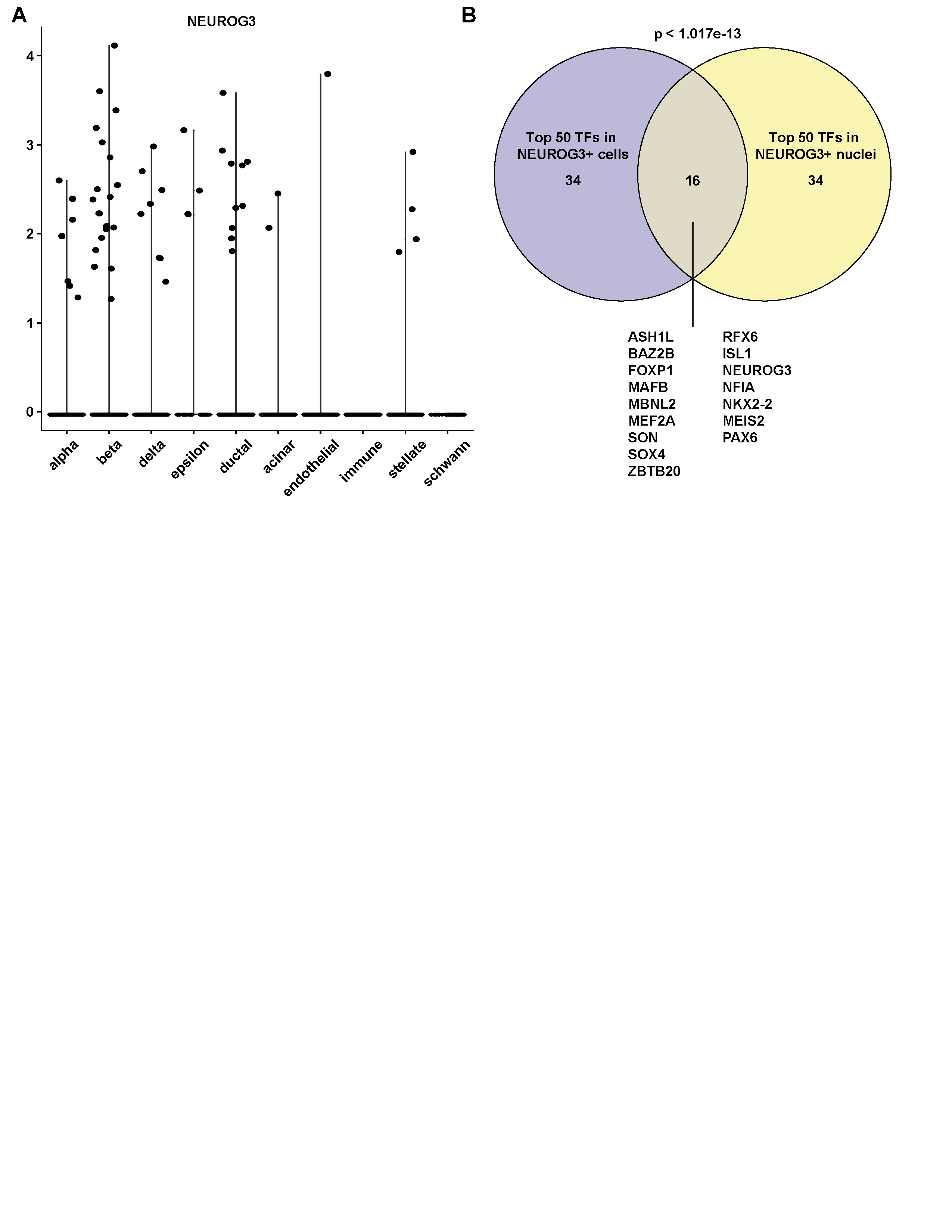

Supplement: Supplementary Figure 5 — Validation of the integrated single-cell RNA-seq dataset with a single-nucleus RNA-seq dataset. (A) Scatter plot exhibits the expression levels of NEUROG3 in different populations. (B) Venn diagram demonstrates the overlap of the top 50 most highly expressed transcription factors in the datasets. The significance of the overlap is calculated based on hypergeometric distribution. [file Image_5.tif]
