# Supplementary material for: Gene Signatures of NEUROGENIN3+ Endocrine Progenitor Cells in the Human Pancreas
Source: Front Endocrinol (Lausanne). 2021 Sep 8;12:736286. doi: 10.3389/fendo.2021.736286 (PMC8456125; doi:10.3389/fendo.2021.736286)
Supplement: Supplementary file 6 [file Table_1.docx]

**Table S1. Summary of four single-cell RNA-seq datasets. Related to Figure 1.**

| Studies | | Enge | Segerstolpe | Wang_C1 | Wang_C1HT |
| --- | --- | --- | --- | --- | --- |
| Single cell platform | | Plate based Smart-seq2 | Plate based Smart-seq2 | Fluidigm C1 | Fluidigm C1HT |
| Median reads/cell | | 510,388 | 312,402 | 1,820,102 | 378,824 |
| Median genes/cell | | 3,244 | 6,186 | 6,102 | 4,127 |
| Number of cells | total | 2,504 | 2,470 | 1283 | 4917 |
|  | alpha | 1,016 | 1,038 | 369 | 1,718 |
|  | beta | 441 | 310 | 296 | 801 |
|  | delta | 71 | 101 | 35 | 151 |
|  | epsilon | 8 | 7 | 1 | 19 |
|  | pp | 20 | 201 | 33 | 32 |
|  | ductal | 418 | 436 | 244 | 736 |
|  | acinar | 405 | 208 | 139 | 681 |
|  | endothelial | 13 | 25 | 31 | 125 |
|  | fibroblast | 60 | 67 | 103 | 180 |
|  | immune | 1 | 19 | 12 | 23 |
|  | doublets | 51 | 58 | 28 | 451 |
