# Supplementary material for: Gene Signatures of NEUROGENIN3+ Endocrine Progenitor Cells in the Human Pancreas
Source: Front Endocrinol (Lausanne). 2021 Sep 8;12:736286. doi: 10.3389/fendo.2021.736286 (PMC8456125; doi:10.3389/fendo.2021.736286)
Supplement: Supplementary file 7 [file Table_2.docx]

**Table S2. Summary of metadata associated with the 12 NEUROG3+ cells. Related to Figure 1.**

| Cell ID | Source | Cell Type Label | Original Cell Type Label | Total Reads | Number of Genes Detected | Donor Label | Donor Type | Age |
| --- | --- | --- | --- | --- | --- | --- | --- | --- |
| GSM2172811_1000101501.C9 | Enge | alpha | unknown | 170,560 | 2278 | 1 | Control | 1 month |
| GSM2172813_1000101501.H6 | Enge | beta | beta | 444,958 | 2135 | 1 | Control | 1 month |
| GSM2173826_1000101503.G11 | Enge | alpha | unknown | 622,229 | 1746 | 1 | Control | 1 month |
| GSM2174339_1000102803.C6 | Enge | epsilon | unknown | 254,152 | 2826 | 5 | Control | 22 years |
| ERR1630516 | Segerstolpe | beta | doublets | 690,722 | 7929 | HP1504101 | T2D | 57 years |
| ERR1630896 | Segerstolpe | epsilon | epsilon | 327,638 | 3509 | HP1504901 | Control | 23 years |
| ERR1631896 | Segerstolpe | immune | unknown | 239,376 | 6900 | HP1507101 | Control | 22 years |
| ERR1632118 | Segerstolpe | immune | immune | 588,622 | 4966 | HP1508501 | T2D | 37 years |
| ERR1633014 | Segerstolpe | alpha | alpha | 254,691 | 7215 | HP1525301 | T2D | 52 years |
| reads.39986 | Wang_C1 | beta | beta | 1,376,913 | 8507 | ICRH85 | Control | 18 days |
| sc50199 | Wang_C1HT | ductal | unknown | 700,281 | 4464 | HPAP002 | T1D | 26 years |
| sc64365 | Wang_C1HT | epsilon | unknown | 230,998 | 3174 | HPAP012 | Control | 18 years |
