# Supplementary material for: Gene Signatures of NEUROGENIN3+ Endocrine Progenitor Cells in the Human Pancreas
Source: Front Endocrinol (Lausanne). 2021 Sep 8;12:736286. doi: 10.3389/fendo.2021.736286 (PMC8456125; doi:10.3389/fendo.2021.736286)
Supplement: Supplementary file 8 [file Table_3.docx]

**Table S3. Differentially expressed transcripts in the NEUROG3+ cells. Related to Figure 2.**

|  | Log2FC | AveExpr | t | P.Value | adj.P.Val | B |
| --- | --- | --- | --- | --- | --- | --- |
| NEUROG3 | 2.71826783 | 1.61483911 | 162.622759 | 0 | 0 | 6170.10587 |
| PROX1 | 2.56239373 | 3.06025864 | 3.65548179 | 0.00025803 | 0.04525821 | 0.00541833 |
| PPIH | 2.33674608 | 2.49548808 | 4.31454635 | 1.61E-05 | 0.00381827 | 2.57343478 |
| TMCC2 | 2.3218128 | 2.04786434 | 5.52388773 | 3.40E-08 | 1.17E-05 | 8.38512382 |
| GLYCTK | 1.9070131 | 2.05098646 | 4.68676805 | 2.81E-06 | 0.00076116 | 4.21072499 |
| GHRL | 1.88734837 | 1.77741412 | 8.24470637 | 1.87E-16 | 1.60E-13 | 26.6374386 |
| AZGP1 | 1.88113182 | 2.28881324 | 4.12434769 | 3.75E-05 | 0.00786351 | 1.78888305 |
| TM4SF5 | 1.84172427 | 1.74067848 | 7.68402121 | 1.69E-14 | 1.20E-11 | 22.2923936 |
| ATP2C2 | 1.78710852 | 1.91213881 | 5.10128614 | 3.44E-07 | 0.00010734 | 6.19267497 |
| RSPH9 | 1.72341451 | 1.83667366 | 5.70456185 | 1.20E-08 | 4.54E-06 | 9.3753463 |
| C6orf192 | 1.65094933 | 1.90084467 | 4.53158906 | 5.92E-06 | 0.0015456 | 3.51174995 |
| POLG2 | 1.64056626 | 2.17254259 | 3.73425077 | 0.00018933 | 0.03418949 | 0.2900598 |
| CSMD1 | 1.60867664 | 1.75846348 | 6.43434223 | 1.30E-10 | 6.17E-08 | 13.6969625 |
| VTN | 1.56240113 | 2.01125983 | 3.80089377 | 0.00014504 | 0.02664096 | 0.53560765 |
| ACCS | 1.53577569 | 1.98444285 | 4.17458675 | 3.01E-05 | 0.00644277 | 1.99268998 |
| C2orf56 | 1.51899856 | 1.86157894 | 4.62128924 | 3.86E-06 | 0.00102022 | 3.91293044 |
| IL1RAP | 1.49957705 | 1.97695849 | 4.13870356 | 3.52E-05 | 0.00746129 | 1.84687022 |
| FAM13C | 1.45412242 | 1.91827635 | 4.26302855 | 2.04E-05 | 0.0046336 | 2.35744935 |
| RHBDL1 | 1.44282565 | 1.80859345 | 5.43312905 | 5.67E-08 | 1.90E-05 | 7.89965107 |
| DOCK8 | 1.41361486 | 1.7950223 | 5.29198119 | 1.24E-07 | 4.04E-05 | 7.16052868 |
| ECEL1 | 1.41007612 | 1.73807929 | 5.84171671 | 5.33E-09 | 2.15E-06 | 10.1481866 |
| MEP1B | 1.39999401 | 1.68527182 | 8.08779067 | 6.79E-16 | 5.38E-13 | 25.3909978 |
| PCK1 | 1.39819522 | 1.7350289 | 5.13922592 | 2.81E-07 | 8.91E-05 | 6.38241991 |
| HLA-DPA1 | 1.38326063 | 1.79159214 | 4.95769287 | 7.25E-07 | 0.00021257 | 5.48719846 |
| APOA1 | 1.33626158 | 1.62707272 | 17.3516779 | 1.83E-66 | 7.11E-63 | 138.66433 |
| OPRK1 | 1.22271796 | 1.6277483 | 16.4782292 | 3.31E-60 | 1.09E-56 | 124.608958 |
| H19 | 1.20166445 | 1.74291453 | 4.685655 | 2.83E-06 | 0.00076116 | 4.20562802 |
| ASGR1 | 1.15531417 | 1.72677719 | 5.55512549 | 2.85E-08 | 1.01E-05 | 8.554065 |
| DCDC5 | 1.15395242 | 1.70773786 | 5.44298622 | 5.36E-08 | 1.81E-05 | 7.95199063 |
| C20orf118 | 1.13435155 | 1.64466951 | 9.92744521 | 4.05E-23 | 5.10E-20 | 41.484511 |
| ZNF225 | 1.12108988 | 1.76274248 | 4.37437684 | 1.23E-05 | 0.00299158 | 2.82751432 |
| APOC3 | 1.08855164 | 1.61630149 | 26.5422655 | 5.05E-150 | 5.40E-146 | 326.5065 |
| M1 | 1.05026227 | 1.79566586 | 4.40839902 | 1.05E-05 | 0.00260335 | 2.97354927 |
| ZNF323 | 1.0358941 | 1.8001242 | 3.63209931 | 0.00028254 | 0.04895662 | -0.0779124 |
| PDE2A | 1.03577155 | 1.66701138 | 6.85887521 | 7.35E-12 | 4.09E-09 | 16.4479793 |
| DCHS1 | 1.02361292 | 1.78974674 | 3.98943225 | 6.67E-05 | 0.01309615 | 1.25373532 |
| OLFML3 | 1.012443 | 1.69594233 | 5.27271478 | 1.37E-07 | 4.45E-05 | 7.06114014 |
| TAS2R5 | 1.01021405 | 1.64077085 | 9.15926441 | 6.26E-20 | 6.70E-17 | 34.3714047 |
| TF | 0.99513653 | 1.65716676 | 7.06592417 | 1.70E-12 | 1.00E-09 | 17.85281 |
| NRG1 | 0.98874194 | 1.7953591 | 4.13366298 | 3.60E-05 | 0.00758906 | 1.82648709 |
| LOC102724571 | 0.97355299 | 1.63695689 | 8.56103653 | 1.28E-17 | 1.22E-14 | 29.2219361 |
| LOC340512 | 0.96441921 | 1.62631981 | 11.3064727 | 1.85E-29 | 3.43E-26 | 55.6586188 |
| KRTAP2-3 | 0.95542986 | 1.69122873 | 4.73452644 | 2.23E-06 | 0.00061466 | 4.43055829 |
| HSF2BP | 0.94849056 | 1.66339237 | 6.55864464 | 5.70E-11 | 2.84E-08 | 14.484425 |
| LOC105372797 | 0.94222593 | 1.62483309 | 11.8224426 | 4.90E-32 | 1.10E-28 | 61.4231235 |
| ANGPTL3 | 0.93956225 | 1.71142755 | 4.53513116 | 5.82E-06 | 0.00152926 | 3.52744335 |
| LRRC37A4 | 0.93955305 | 1.75773282 | 4.28539102 | 1.84E-05 | 0.00427458 | 2.45088526 |
| SHLD3 | 0.93153576 | 1.69365401 | 5.5778321 | 2.50E-08 | 8.91E-06 | 8.67746182 |
| LOC107985066 | 0.92625411 | 1.64846919 | 7.8398308 | 4.98E-15 | 3.67E-12 | 23.4695454 |
| MEGF6 | 0.90866285 | 1.69939726 | 5.03258898 | 4.92E-07 | 0.00015053 | 5.85266459 |
| RGCC | 0.90813115 | 1.74973126 | 4.0102288 | 6.11E-05 | 0.01210723 | 1.33506938 |
| EPHB1 | 0.89889745 | 1.65863149 | 6.4022751 | 1.60E-10 | 7.53E-08 | 13.4962396 |
| LOC646719 | 0.89345715 | 1.75174941 | 3.6874152 | 0.00022775 | 0.04027846 | 0.12008434 |
| LOC100506804 | 0.87941784 | 1.67250559 | 5.33376167 | 9.83E-08 | 3.26E-05 | 7.37729754 |
| FAM95A | 0.87075371 | 1.61725533 | 21.0063048 | 6.91E-96 | 4.93E-92 | 204.784701 |
| C19orf38 | 0.85887604 | 1.66764365 | 5.64197758 | 1.73E-08 | 6.32E-06 | 9.0287561 |
| TXLNG2P | 0.84877148 | 1.73436847 | 3.82350704 | 0.00013237 | 0.02441925 | 0.61991083 |
| IL12A | 0.8367513 | 1.64592878 | 6.96265164 | 3.55E-12 | 2.03E-09 | 17.1469339 |
| LOC105371047 | 0.82820235 | 1.61547946 | 19.518898 | 2.74E-83 | 1.30E-79 | 176.466456 |
| LOC107984210 | 0.82336892 | 1.65001867 | 6.25860159 | 4.05E-10 | 1.86E-07 | 12.6091314 |
| BMP7 | 0.81192057 | 1.64064848 | 7.67732076 | 1.78E-14 | 1.25E-11 | 22.2422948 |
| LOC105377086 | 0.80876387 | 1.62202247 | 10.3954376 | 3.50E-25 | 4.84E-22 | 46.0929488 |
| SCN5A | 0.80642649 | 1.63764343 | 8.35152502 | 7.64E-17 | 6.81E-14 | 27.49945 |
| BHMT | 0.79932995 | 1.63675226 | 8.27986898 | 1.39E-16 | 1.22E-13 | 26.919988 |
| TMEM190 | 0.78350882 | 1.63774262 | 8.17240546 | 3.39E-16 | 2.74E-13 | 26.0601903 |
| IL17D | 0.76952407 | 1.71252586 | 3.95427776 | 7.73E-05 | 0.0149696 | 1.11720669 |
| LOC101928152 | 0.7670584 | 1.6820019 | 4.42822523 | 9.60E-06 | 0.00240342 | 3.05916993 |
| LOC105372754 | 0.76032095 | 1.68112708 | 4.51926416 | 6.28E-06 | 0.00162823 | 3.45723917 |
| GRAMD2A | 0.75967925 | 1.66476919 | 5.52825873 | 3.32E-08 | 1.15E-05 | 8.4087063 |
| LOC107986477 | 0.75647698 | 1.63646458 | 7.30101479 | 3.08E-13 | 1.85E-10 | 19.4980236 |
| C19orf45 | 0.74166959 | 1.65050313 | 5.83648758 | 5.50E-09 | 2.20E-06 | 10.1183872 |
| LOC105373021 | 0.73269525 | 1.62340108 | 10.9654172 | 8.09E-28 | 1.39E-24 | 51.9858347 |
| PHGR1 | 0.72840742 | 1.71435306 | 4.17785448 | 2.97E-05 | 0.006383 | 2.00603149 |
| NPSR1-AS1 | 0.72583479 | 1.63627152 | 6.63615123 | 3.39E-11 | 1.73E-08 | 14.9829925 |
| WDR67 | 0.72300341 | 1.67552104 | 4.06514767 | 4.84E-05 | 0.00981164 | 1.55187987 |
| LOC254128 | 0.71819529 | 1.67422782 | 4.37481427 | 1.23E-05 | 0.00299158 | 2.82938477 |
| SNRK-AS1 | 0.70737345 | 1.63687279 | 6.81349305 | 1.01E-11 | 5.53E-09 | 16.1455883 |
| KCNQ5 | 0.7033526 | 1.63134764 | 8.17256189 | 3.39E-16 | 2.74E-13 | 26.0614339 |
| LINC00538 | 0.70029341 | 1.61917438 | 11.7219178 | 1.59E-31 | 3.24E-28 | 60.2804254 |
| MROH7 | 0.6903214 | 1.66721699 | 4.80628249 | 1.56E-06 | 0.0004393 | 4.76502408 |
| AKR1E2 | 0.68796384 | 1.69981131 | 3.92923975 | 8.58E-05 | 0.01639186 | 1.02070166 |
| LOC100130522 | 0.68755205 | 1.64464936 | 6.14761265 | 8.17E-10 | 3.64E-07 | 11.9375074 |
| FAM5C | 0.68541662 | 1.61499334 | 16.7433843 | 4.49E-62 | 1.60E-58 | 128.803413 |
| LOC105375624 | 0.68060377 | 1.62896807 | 7.99373397 | 1.46E-15 | 1.11E-12 | 24.6551972 |
| FAM198A | 0.67802967 | 1.67376238 | 4.14270681 | 3.46E-05 | 0.0073689 | 1.86307618 |
| LINC00852 | 0.67462368 | 1.64739761 | 5.95293478 | 2.72E-09 | 1.17E-06 | 10.788264 |
| C1orf170 | 0.67382248 | 1.6250315 | 9.93144029 | 3.90E-23 | 5.05E-20 | 41.5229719 |
| LOC100130950 | 0.66539046 | 1.6726757 | 4.30237717 | 1.71E-05 | 0.0039897 | 2.52218304 |
| KCNIP2 | 0.66363139 | 1.66281521 | 4.76142696 | 1.95E-06 | 0.00054533 | 4.55535896 |
| NFE2 | 0.66063981 | 1.62052455 | 11.6014594 | 6.41E-31 | 1.25E-27 | 58.9236319 |
| LIPJ | 0.6367285 | 1.66582859 | 4.19118233 | 2.80E-05 | 0.00614342 | 2.06055425 |
| COL11A2 | 0.63512776 | 1.62949623 | 7.72284431 | 1.25E-14 | 9.05E-12 | 22.5835215 |
| GPR133 | 0.63193728 | 1.64174195 | 5.90056716 | 3.74E-09 | 1.54E-06 | 10.4853872 |
| XRCC2 | 0.62054352 | 1.64603042 | 5.53378285 | 3.21E-08 | 1.13E-05 | 8.43853654 |
| ABCA4 | 0.61635 | 1.63744901 | 5.84282559 | 5.29E-09 | 2.15E-06 | 10.1545093 |
| FLJ14186 | 0.61309297 | 1.68139324 | 4.26566195 | 2.01E-05 | 0.00460387 | 2.36842703 |
| LOC728723 | 0.60443671 | 1.66499041 | 4.18560602 | 2.87E-05 | 0.00623202 | 2.03772112 |
| FAM211A | 0.60340063 | 1.65056033 | 4.46518308 | 8.09E-06 | 0.0020363 | 3.21979601 |
| LOC93432 | 0.59747974 | 1.6670602 | 4.07797804 | 4.58E-05 | 0.0093308 | 1.60295554 |
| LOC105369588 | 0.59692257 | 1.62208362 | 9.61068854 | 8.97E-22 | 1.07E-18 | 38.4833733 |
| PGAM2 | 0.59601606 | 1.67296126 | 3.62262595 | 0.00029308 | 0.0497755 | -0.1115218 |
| HIST2H2BC | 0.59429238 | 1.62798422 | 9.08613785 | 1.22E-19 | 1.25E-16 | 33.7235946 |
| LOC105371219 | 0.59204518 | 1.64261743 | 4.97605528 | 6.60E-07 | 0.00019611 | 5.57629599 |
| KCNG1 | 0.59066532 | 1.68883752 | 3.71333494 | 0.00020567 | 0.03682908 | 0.21388781 |
| ABRA | 0.58483646 | 1.62556499 | 7.64154522 | 2.34E-14 | 1.59E-11 | 21.9755339 |
| SNORD71 | 0.58009305 | 1.62244574 | 9.63741475 | 6.93E-22 | 8.47E-19 | 38.7329064 |
| LOC100527964 | 0.57875136 | 1.66121143 | 3.93715827 | 8.30E-05 | 0.01600416 | 1.05115627 |
| CXorf58 | 0.57754265 | 1.6309565 | 6.7918663 | 1.17E-11 | 6.34E-09 | 16.0021842 |
| CLDN5 | 0.57288182 | 1.65885998 | 4.63696978 | 3.58E-06 | 0.00095185 | 3.98386497 |
| CALCR | 0.56791466 | 1.63482122 | 5.62957705 | 1.86E-08 | 6.73E-06 | 8.9605331 |
| LMOD3 | 0.5629917 | 1.69570797 | 3.63655057 | 0.00027771 | 0.04831545 | -0.06209 |
| PURPL | 0.55793379 | 1.61600675 | 15.3678185 | 1.09E-52 | 3.10E-49 | 107.733462 |
| COL4A5 | 0.55744927 | 1.64301676 | 5.19567854 | 2.08E-07 | 6.69E-05 | 6.66733972 |
| GJB7 | 0.55475512 | 1.65971325 | 3.89283828 | 9.97E-05 | 0.01871988 | 0.88148796 |
| HLA-DQA2 | 0.54754987 | 1.63305256 | 5.7746271 | 7.94E-09 | 3.06E-06 | 9.7678717 |
| PCSK6 | 0.54710483 | 1.65551581 | 4.19371225 | 2.77E-05 | 0.0061067 | 2.07092339 |
| ST8SIA6 | 0.53955893 | 1.62272234 | 7.54173366 | 5.04E-14 | 3.37E-11 | 21.237797 |
| FLJ30403 | 0.53865887 | 1.6623067 | 3.83352747 | 0.0001271 | 0.0235481 | 0.65742681 |
| LOC100507250 | 0.53825951 | 1.64912614 | 4.11438769 | 3.91E-05 | 0.00817018 | 1.74876996 |
| HIST1H1T | 0.5366538 | 1.62176557 | 8.55789493 | 1.32E-17 | 1.23E-14 | 29.1957971 |
| SAMSN1 | 0.53175515 | 1.65898743 | 3.66923765 | 0.00024455 | 0.043071 | 0.05469071 |
| LOC283693 | 0.53111803 | 1.63173433 | 6.66437595 | 2.80E-11 | 1.46E-08 | 15.1659921 |
| CACNA2D4 | 0.53073156 | 1.63096257 | 6.06434684 | 1.37E-09 | 5.94E-07 | 11.441472 |
| HIST1H3C | 0.52935144 | 1.61926245 | 8.58804021 | 1.02E-17 | 9.90E-15 | 29.4470038 |
| LOC105374071 | 0.52765711 | 1.62323726 | 8.0064377 | 1.31E-15 | 1.02E-12 | 24.7540817 |
| C2orf40 | 0.51803775 | 1.65017392 | 4.21542157 | 2.52E-05 | 0.00563596 | 2.16015776 |
| LOC105374898 | 0.5163177 | 1.62223962 | 7.64858283 | 2.22E-14 | 1.53E-11 | 22.0279127 |
| PHACTR3 | 0.51128041 | 1.65011404 | 3.84000247 | 0.0001238 | 0.02303623 | 0.68172097 |
| PTPRD-AS1 | 0.51100645 | 1.63806847 | 4.95855916 | 7.22E-07 | 0.00021257 | 5.4913945 |
| COL2A1 | 0.51081022 | 1.62547502 | 6.46959974 | 1.03E-10 | 5.00E-08 | 13.9188032 |
| NBEAP1 | 0.50929502 | 1.62994927 | 6.55256665 | 5.94E-11 | 2.92E-08 | 14.4455733 |
| FAM217A | 0.50751059 | 1.62422355 | 7.40896128 | 1.38E-13 | 8.54E-11 | 20.2712966 |
| HIST1H2BE | 0.50523657 | 1.63737594 | 6.55923417 | 5.68E-11 | 2.84E-08 | 14.4881953 |
| NOTUM | 0.50488971 | 1.61821276 | 11.1469829 | 1.10E-28 | 1.96E-25 | 53.927445 |
| C8orf51 | 0.49680767 | 1.62811835 | 5.48953935 | 4.13E-08 | 1.40E-05 | 8.20045256 |
| MFAP4 | 0.49096372 | 1.6448158 | 5.52115516 | 3.45E-08 | 1.18E-05 | 8.37039048 |
| LINC01484 | 0.48764904 | 1.66356477 | 3.77870121 | 0.00015857 | 0.02887891 | 0.45335763 |
| MSLN | 0.47729652 | 1.62389487 | 6.68685295 | 2.40E-11 | 1.27E-08 | 15.3122758 |
| SLED1 | 0.47376816 | 1.61834172 | 9.21118683 | 3.88E-20 | 4.26E-17 | 34.8344711 |
| C11orf82 | 0.4677004 | 1.62609523 | 5.58652473 | 2.38E-08 | 8.55E-06 | 8.72483347 |
| SLC7A9 | 0.46708303 | 1.64733821 | 3.62608135 | 0.0002892 | 0.04931106 | -0.0992729 |
| FLJ35282 | 0.46569271 | 1.61493521 | 14.1087149 | 9.08E-45 | 2.16E-41 | 89.9572888 |
| MIR29B2 | 0.4655021 | 1.63960062 | 5.06046705 | 4.26E-07 | 0.00013108 | 5.99009171 |
| LINC00668 | 0.46227922 | 1.62183762 | 7.43949382 | 1.09E-13 | 6.89E-11 | 20.4920519 |
| LOC100506012 | 0.46083659 | 1.63414636 | 4.80891709 | 1.54E-06 | 0.00043644 | 4.77739966 |
| PNPLA3 | 0.45983356 | 1.64189897 | 4.32939663 | 1.51E-05 | 0.0036303 | 2.63617363 |
| LINC01915 | 0.45832622 | 1.62573772 | 6.39513962 | 1.68E-10 | 7.80E-08 | 13.4517106 |
| ATP2B3 | 0.45595843 | 1.63181381 | 5.07071474 | 4.03E-07 | 0.00012513 | 6.04079833 |
| CR2 | 0.4519533 | 1.62117348 | 7.00234121 | 2.68E-12 | 1.55E-09 | 17.4169981 |
| LOC158435 | 0.44973868 | 1.62030626 | 7.06515171 | 1.71E-12 | 1.00E-09 | 17.847492 |
| MIR6719 | 0.44787931 | 1.6151396 | 14.4850523 | 4.55E-47 | 1.14E-43 | 95.1181914 |
| STX1B | 0.44299973 | 1.6408994 | 4.8780871 | 1.09E-06 | 0.00031672 | 5.10472826 |
| LOC100507050 | 0.43616783 | 1.61716737 | 10.5894418 | 4.59E-26 | 7.02E-23 | 48.0642219 |
| LINC01098 | 0.43083491 | 1.61977249 | 7.3676111 | 1.88E-13 | 1.15E-10 | 19.973759 |
| SNORA50 | 0.43066216 | 1.62341793 | 6.89148136 | 5.86E-12 | 3.30E-09 | 16.6664683 |
| VAV1 | 0.42923597 | 1.63886805 | 4.1877682 | 2.84E-05 | 0.00620456 | 2.04657091 |
| CASC17 | 0.42676868 | 1.61458814 | 16.2203763 | 2.04E-58 | 6.23E-55 | 120.59076 |
| SLMO1 | 0.42596479 | 1.62417435 | 6.11630651 | 9.94E-10 | 4.38E-07 | 11.7502213 |
| GALNT16 | 0.42469905 | 1.64262817 | 4.03370853 | 5.53E-05 | 0.01106249 | 1.42740372 |
| ITIH1 | 0.41333995 | 1.62118855 | 6.70977525 | 2.06E-11 | 1.10E-08 | 15.4619603 |
| BNC1 | 0.40822859 | 1.61639025 | 10.5349031 | 8.16E-26 | 1.20E-22 | 47.5064552 |
| HCG20 | 0.40681215 | 1.62644394 | 5.83352969 | 5.60E-09 | 2.22E-06 | 10.1015426 |
| SCARNA18 | 0.40027526 | 1.63637238 | 4.3186889 | 1.58E-05 | 0.00376826 | 2.59091447 |
| C9orf139 | 0.40003845 | 1.63325304 | 4.35862746 | 1.32E-05 | 0.00319654 | 2.76029413 |
| CERS6-AS1 | 0.39848483 | 1.63269953 | 5.32424199 | 1.04E-07 | 3.41E-05 | 7.32775768 |
| LOC100129827 | 0.39692805 | 1.6172687 | 9.24235785 | 2.91E-20 | 3.27E-17 | 35.1137031 |
| ITGA2B | 0.39560975 | 1.63804046 | 3.62617876 | 0.00028909 | 0.04931106 | -0.0989275 |
| ADAM2 | 0.37760244 | 1.62134163 | 5.92607584 | 3.21E-09 | 1.35E-06 | 10.632589 |
| GFAP | 0.37165719 | 1.62786101 | 5.18684334 | 2.18E-07 | 6.97E-05 | 6.62254361 |
| MEGF10 | 0.36497377 | 1.63309364 | 3.78813144 | 0.00015268 | 0.02792509 | 0.48824926 |
| SNORA68 | 0.36477537 | 1.6380877 | 4.26624157 | 2.01E-05 | 0.00460387 | 2.37084419 |
| CRISPLD1 | 0.357474 | 1.6222304 | 5.01870596 | 5.29E-07 | 0.00016064 | 5.78450881 |
| ASGR2 | 0.35498322 | 1.61468312 | 11.7838599 | 7.71E-32 | 1.65E-28 | 60.98342 |
| LINC01445 | 0.34803591 | 1.61345101 | 14.5931686 | 9.69E-48 | 2.59E-44 | 96.6249064 |
| SNORD116-29 | 0.34802052 | 1.63626775 | 5.11925582 | 3.12E-07 | 9.83E-05 | 6.28237088 |
| GP5 | 0.34294099 | 1.62188635 | 5.78428558 | 7.50E-09 | 2.92E-06 | 9.8223545 |
| SFTPC | 0.33739982 | 1.61992922 | 6.07840439 | 1.26E-09 | 5.50E-07 | 11.5247454 |
| ESPN | 0.33145593 | 1.62388343 | 5.69379832 | 1.28E-08 | 4.80E-06 | 9.31546769 |
| LOC101928205 | 0.3297543 | 1.61385231 | 10.7408601 | 9.18E-27 | 1.46E-23 | 49.6275252 |
| BK250D10.8 | 0.32777881 | 1.61295507 | 20.9807954 | 1.15E-95 | 7.06E-92 | 204.282987 |
| HIST1H3E | 0.32604668 | 1.62426011 | 4.50463131 | 6.72E-06 | 0.00172342 | 3.39271267 |
| ERVW-1 | 0.32038937 | 1.62844732 | 4.10354809 | 4.10E-05 | 0.0085205 | 1.70522421 |
| LINC00589 | 0.31533093 | 1.62178209 | 4.83069904 | 1.38E-06 | 0.000394 | 4.87997511 |
| BLK | 0.30452653 | 1.61794937 | 6.18929298 | 6.28E-10 | 2.83E-07 | 12.1883283 |
| CHL1-AS1 | 0.29962466 | 1.61799515 | 6.65984457 | 2.89E-11 | 1.49E-08 | 15.1365603 |
| TMEM229A | 0.29195976 | 1.62375695 | 4.48855754 | 7.25E-06 | 0.00184734 | 3.32207235 |
| LOC105373881 | 0.29139306 | 1.61587655 | 7.8546464 | 4.42E-15 | 3.32E-12 | 23.5826932 |
| LOC107987437 | 0.28946828 | 1.62117209 | 5.66279304 | 1.53E-08 | 5.65E-06 | 9.14361005 |
| LOC105375626 | 0.28776792 | 1.6123389 | 33.7156411 | 5.10E-236 | 1.09E-231 | 519.851419 |
| NBPF24 | 0.28337911 | 1.63096934 | 5.92716128 | 3.18E-09 | 1.35E-06 | 10.6388667 |
| KRTAP12-2 | 0.28247669 | 1.62160254 | 4.84566127 | 1.28E-06 | 0.00036796 | 4.95070244 |
| LOC643339 | 0.27517169 | 1.62096475 | 4.4219652 | 9.88E-06 | 0.00245961 | 3.03209428 |
| LOC100289092 | 0.27359913 | 1.61567456 | 10.4227792 | 2.64E-25 | 3.76E-22 | 46.3686085 |
| RAPGEF4-AS1 | 0.27354963 | 1.62419248 | 4.28462604 | 1.85E-05 | 0.00427458 | 2.44768094 |
| SNORD25 | 0.27074289 | 1.63555145 | 3.62641932 | 0.00028882 | 0.04931106 | -0.0980743 |
| CRAT37 | 0.2680552 | 1.62825006 | 4.08014398 | 4.54E-05 | 0.00931858 | 1.6115936 |
| LOC100506334 | 0.25953688 | 1.62249352 | 4.18068207 | 2.93E-05 | 0.00633612 | 2.01758437 |
| LOC105377456 | 0.25214984 | 1.61530531 | 8.19214401 | 2.88E-16 | 2.42E-13 | 26.2172847 |
| LOC107986710 | 0.24398657 | 1.62052309 | 4.30933937 | 1.65E-05 | 0.00388762 | 2.55148742 |
| LINC00479 | 0.24217902 | 1.62196788 | 4.00588353 | 6.22E-05 | 0.0122748 | 1.31804046 |
| ADCYAP1R1 | 0.24163777 | 1.61742531 | 5.90316019 | 3.68E-09 | 1.53E-06 | 10.5003218 |
| PIK3R5 | 0.24072126 | 1.61982344 | 3.96094723 | 7.52E-05 | 0.01462434 | 1.14301616 |
| LINC02380 | 0.23453779 | 1.61259115 | 19.630154 | 3.35E-84 | 1.79E-80 | 178.518363 |
| SPATA31D1 | 0.23396966 | 1.6355825 | 3.90542529 | 9.47E-05 | 0.01789607 | 0.92947962 |
| FARSA-AS1 | 0.22909063 | 1.61798783 | 5.77144422 | 8.10E-09 | 3.09E-06 | 9.7499371 |
| MIR2052HG | 0.21592938 | 1.62178247 | 4.09989612 | 4.17E-05 | 0.00861403 | 1.69057898 |
| SNORD114-27 | 0.21464806 | 1.61439137 | 9.31365908 | 1.50E-20 | 1.73E-17 | 35.755909 |
| MIR4519 | 0.20819986 | 1.62068125 | 4.39260336 | 1.13E-05 | 0.00278345 | 2.90560871 |
| LOC728437 | 0.20750982 | 1.61884634 | 4.02799349 | 5.67E-05 | 0.01128163 | 1.40487979 |
| LOC647323 | 0.20675186 | 1.62009458 | 3.64293502 | 0.00027092 | 0.04732623 | -0.0393623 |
| TRIM42 | 0.20579507 | 1.61392151 | 8.65172495 | 5.86E-18 | 5.83E-15 | 29.9805618 |
| JARID2-AS1 | 0.20459553 | 1.6196936 | 3.87613078 | 0.00010682 | 0.01996271 | 0.81802433 |
| RPRM | 0.20457728 | 1.61866177 | 4.079395 | 4.55E-05 | 0.00931858 | 1.60860605 |
| RNU5B-1 | 0.20390417 | 1.61759241 | 4.66171256 | 3.18E-06 | 0.00084976 | 4.09628112 |
| FBXO47 | 0.20282519 | 1.61744404 | 4.86959143 | 1.14E-06 | 0.00032838 | 5.0642742 |
| OR4F3 | 0.19636305 | 1.61948489 | 3.62789499 | 0.00028718 | 0.04931106 | -0.0928392 |
| LINC01901 | 0.17730622 | 1.62008017 | 3.93579882 | 8.35E-05 | 0.01602272 | 1.04592349 |
| LOC105370455 | 0.17086848 | 1.61633568 | 4.20678065 | 2.61E-05 | 0.00582484 | 2.12458495 |
| LOC105369395 | 0.17044874 | 1.61575034 | 4.75530434 | 2.01E-06 | 0.00055843 | 4.52689217 |
| LINC00635 | 0.16962471 | 1.61852763 | 3.7015487 | 0.00021545 | 0.03841984 | 0.17115221 |
| SASH3 | 0.16476866 | 1.61473133 | 6.44751687 | 1.19E-10 | 5.72E-08 | 13.7797169 |
| LINC01267 | 0.1647122 | 1.61756961 | 4.0469412 | 5.23E-05 | 0.0105552 | 1.4796781 |
| MIR636 | 0.15584057 | 1.62080573 | 3.7580449 | 0.00017223 | 0.03123317 | 0.37723279 |
| LAIR2 | 0.14620412 | 1.61530468 | 3.92590944 | 8.70E-05 | 0.01654613 | 1.00791155 |
| MIR1287 | 0.14067709 | 1.61300421 | 10.8406572 | 3.14E-27 | 5.17E-24 | 50.6697189 |
| LINC01289 | 0.13615093 | 1.61680076 | 4.47526241 | 7.72E-06 | 0.00195424 | 3.26383347 |
| LOC105369205 | 0.13556457 | 1.61695288 | 3.96105071 | 7.51E-05 | 0.01462434 | 1.14341696 |
| ATP2C2-AS1 | 0.13473977 | 1.61667973 | 4.24502314 | 2.21E-05 | 0.0049945 | 2.28257223 |
| KRTAP2-4 | 0.13455224 | 1.61443243 | 5.66455381 | 1.52E-08 | 5.64E-06 | 9.15334478 |
| DUXAP9 | 0.13306405 | 1.62052607 | 3.72720999 | 0.00019469 | 0.03500942 | 0.26437081 |
| MIR500A | 0.12726806 | 1.61298491 | 7.5052044 | 6.66E-14 | 4.32E-11 | 20.9701946 |
| NPIPB12 | 0.09131364 | 1.61326569 | 8.47414779 | 2.70E-17 | 2.46E-14 | 28.5024821 |
| LOC107987137 | 0.08308591 | 1.61390304 | 4.20086179 | 2.68E-05 | 0.00594799 | 2.10026028 |
| IFNA17 | 0.08300575 | 1.61342937 | 4.32632983 | 1.53E-05 | 0.00366052 | 2.62319952 |
| LOC105378378 | 0.08292277 | 1.61346081 | 5.00398248 | 5.71E-07 | 0.00017096 | 5.71243165 |
| LOC105377355 | 0.082788 | 1.61262654 | 7.51613912 | 6.13E-14 | 4.04E-11 | 21.0501644 |
| LINC01007 | 0.08263637 | 1.61247581 | 7.46472083 | 9.05E-14 | 5.78E-11 | 20.6751245 |
| TAAR6 | 0.08253314 | 1.6122028 | 27.6228021 | 7.14E-162 | 1.02E-157 | 353.151686 |
| MIR4254 | 0.08243303 | 1.61225915 | 17.9707312 | 4.40E-71 | 1.88E-67 | 149.038807 |
| LOC105375768 | 0.08193157 | 1.61297174 | 4.5076851 | 6.63E-06 | 0.00170909 | 3.40616176 |
| LINC02064 | 0.07353086 | 1.61220387 | 22.8498829 | 1.15E-112 | 9.85E-109 | 242.50574 |
| LINC02088 | 0.07122983 | 1.61389088 | 4.71537903 | 2.45E-06 | 0.00066664 | 4.34215574 |
| LINC01811 | 0.06797804 | 1.61313135 | 4.22789126 | 2.38E-05 | 0.00536118 | 2.21162095 |
| LINC01043 | 0.05319261 | 1.61251854 | 4.03695321 | 5.46E-05 | 0.01096213 | 1.44020574 |
| CDRT15L2 | 0.05308027 | 1.61290943 | 3.90482019 | 9.49E-05 | 0.01789607 | 0.92716898 |
| GSX2 | 0.05256963 | 1.61300641 | 4.72825489 | 2.30E-06 | 0.00062984 | 4.40156366 |
| AGTR2 | 0.03030875 | 1.61225934 | 9.11786712 | 9.15E-20 | 9.55E-17 | 34.0040494 |
| HRH2 | -0.4709265 | 1.63527752 | -5.0163851 | 5.36E-07 | 0.00016144 | 5.77313338 |
| FRZB | -1.0110752 | 1.78686697 | -3.7001193 | 0.00021666 | 0.03847628 | 0.16597839 |
