# Supplementary material for: Gene Signatures of NEUROGENIN3+ Endocrine Progenitor Cells in the Human Pancreas
Source: Front Endocrinol (Lausanne). 2021 Sep 8;12:736286. doi: 10.3389/fendo.2021.736286 (PMC8456125; doi:10.3389/fendo.2021.736286)
Supplement: Supplementary file 9 [file Table_4.docx]

**Table S4. Quantification of the immunostaining. Related to Figure 3.**

|  | ICRH85_slide1 | ICRH85_slide2 | nPOD6407 | HPAP012 |
| --- | --- | --- | --- | --- |
| # of cells | 251886 | 340288 | 603922 | 530931 |
| # of epsilon cells | 3619 | 3840 | 140 | 146 |
| # of other endocrine cells* |  | 99738 |  | 39661 |
| # of exocrine cells |  | 236710 |  | 491124 |
| # NEUROG3+ cells in epsilon | 21 | 29 | 4 | 5 |
| # of NEUROG3+ cells in other endocrine |  | 5 |  | 2 |
| # of NEUROG3+ cells in exocrine |  | 531 |  | 260 |
| NEUROG3+/epsilon (%) | 0.58 | 0.76 | 2.86 | 3.42 |
| NEUROG3+/other endo (%) |  | 0.01 |  | 0.01 |
| NEUROG3+/exocrine (%) |  | 0.22 |  | 0.05 |
| NEUROG3+/total cells (%) |  | 0.17 |  | 0.05 |

* Other endocrine cells correspond to beta (INS+) or alpha (GCG+) or delta (SST+) cells.
