# Supplementary material for: Gene Signatures of NEUROGENIN3+ Endocrine Progenitor Cells in the Human Pancreas
Source: Front Endocrinol (Lausanne). 2021 Sep 8;12:736286. doi: 10.3389/fendo.2021.736286 (PMC8456125; doi:10.3389/fendo.2021.736286)
Supplement: Supplementary file 10 [file Table_5.docx]

**Table S5. Summary of metadata associated with the 55 NEUROG3+ cells in the atlas dataset. Related to Supplementary Figure S4.**

| Cell ID | Cell Type  Label | Total Reads | Number of Genes Detected | Donor Label | Donor Type | Age |
| --- | --- | --- | --- | --- | --- | --- |
| ACTTTCAAGCTATGCT | alpha | 1307 | 794 | st19061908 | Control | 1 day |
| GGGAATGGTCAGCTAT | alpha | 1604 | 1047 | st19061908 | Control | 1 day |
| GGTGAAGGTTCGTTGA | alpha | 1007 | 789 | st19061908 | Control | 1 day |
| GTATTCTAGGCCCTCA | alpha | 3819 | 1845 | st19061908 | Control | 1 day |
| TACCTATTCTGCGTAA | alpha | 3205 | 1768 | st19061908 | Control | 1 day |
| TGACTAGTCTTAGAGC | alpha | 1601 | 908 | st19061908 | Control | 1 day |
| TGGCGCAGTCATTAGC | alpha | 2994 | 1224 | st19061908 | Control | 1 day |
| L1_GGCGACTTCTGCCAGG | beta | 1205 | 830 | IIAM | Control | 1 day |
| L2_ACGGGCTGTACGCTGC | beta | 2503 | 1650 | IIAM | Control | 1 day |
| L2_TATCAGGGTCTAGGTT | beta | 1472 | 936 | IIAM | Control | 1 day |
| L3_ACTGAGTGTTTAGGAA | beta | 609 | 469 | IIAM | Control | 1 day |
| L3_CTCCTAGTCAGGTAAA | beta | 893 | 707 | IIAM | Control | 1 day |
| L3_TGACTTTAGTGCAAGC | beta | 429 | 374 | IIAM | Control | 1 day |
| ACATACGTCCCAAGAT | beta | 3903 | 2057 | st19061908 | Control | 1 day |
| ACCGTAAAGATGTCGG | beta | 1016 | 810 | st19061908 | Control | 1 day |
| AGATCTGAGAGTCGGT | beta | 1413 | 1110 | st19061908 | Control | 1 day |
| AGGCCGTAGCAAATCA | beta | 1648 | 1261 | st19061908 | Control | 1 day |
| AGTAGTCAGTTGTCGT | beta | 848 | 676 | st19061908 | Control | 1 day |
| ATGCGATTCACGATGT | beta | 981 | 726 | st19061908 | Control | 1 day |
| CAACCTCCAAAGCAAT | beta | 1119 | 752 | st19061908 | Control | 1 day |
| CCCAATCTCTGATTCT | beta | 1204 | 843 | st19061908 | Control | 1 day |
| CCTTCCCAGTGTTAGA | beta | 699 | 502 | st19061908 | Control | 1 day |
| CGAGCCATCTCTAAGG | beta | 1011 | 828 | st19061908 | Control | 1 day |
| GTCACAAGTTTCCACC | beta | 1441 | 926 | st19061908 | Control | 1 day |
| TAAACCGTCATATCGG | beta | 2439 | 1136 | st19061908 | Control | 1 day |
| TGGCTGGCACGAAAGC | beta | 829 | 540 | st19061908 | Control | 1 day |
| TTGACTTGTCTAAAGA | beta | 1937 | 1363 | st19061908 | Control | 1 day |
| ACGTCAATCTCTTGAT | delta | 1068 | 680 | st19061908 | Control | 1 day |
| ATCACGACACTGCCAG | delta | 2167 | 1469 | st19061908 | Control | 1 day |
| CATATGGCACTAAGTC | delta | 534 | 450 | st19061908 | Control | 1 day |
| CGCTGGACAGGATTGG | delta | 903 | 554 | st19061908 | Control | 1 day |
| CGGTTAACATAGGATA | delta | 1212 | 966 | st19061908 | Control | 1 day |
| CGTTAGACACGAAATA | delta | 3008 | 953 | st19061908 | Control | 1 day |
| GCTGCAGGTTCAGCGC | delta | 2149 | 583 | st19061908 | Control | 1 day |
| TGAGGGAGTGTTTGGT | delta | 718 | 505 | st19061908 | Control | 1 day |
| CGGCTAGGTCAGAAGC | epsilon | 906 | 599 | st19061908 | Control | 1 day |
| GCTGGGTTCAAGGCTT | epsilon | 882 | 623 | st19061908 | Control | 1 day |
| TGACGGCTCTTAACCT | epsilon | 1228 | 787 | st19061908 | Control | 1 day |
| L2_ATCACGACAGACTCGC | ductal | 1449 | 952 | IIAM | Control | 1 day |
| L3_GATCAGTCACCTCGTT | ductal | 1126 | 830 | IIAM | Control | 1 day |
| AGAGTGGCATCCCACT | ductal | 570 | 500 | st19061908 | Control | 1 day |
| AGGTCATCAAGACGTG | ductal | 1657 | 1247 | st19061908 | Control | 1 day |
| CACCACTTCTGCGGCA | ductal | 1967 | 1371 | st19061908 | Control | 1 day |
| CGTCAGGGTGAGGCTA | ductal | 1121 | 860 | st19061908 | Control | 1 day |
| TAGCCGGAGTGGTCCC | ductal | 641 | 562 | st19061908 | Control | 1 day |
| TCGGTAAAGTCTCGGC | ductal | 1311 | 1025 | st19061908 | Control | 1 day |
| TGGCGCACACGGTGTC | ductal | 670 | 581 | st19061908 | Control | 1 day |
| TTTGCGCTCGAACGGA | ductal | 2191 | 1514 | st19061908 | Control | 1 day |
| CTCGAAATCAGGCAAG | acinar | 940 | 605 | st19061908 | Control | 1 day |
| TCCCGATCATCCGTGG | acinar | 1448 | 882 | st19061908 | Control | 1 day |
| CAACTAGGTCCGAATT | endothelial | 460 | 399 | st19061908 | Control | 1 day |
| CTAGAGTCACATGGGA | stellate | 1678 | 1115 | st19061908 | Control | 1 day |
| GGCTCGACATCGATTG | stellate | 1145 | 792 | st19061908 | Control | 1 day |
| TAGCCGGTCGACAGCC | stellate | 1985 | 1114 | st19061908 | Control | 1 day |
| GGGCACTCAAACGTGG | stellate | 570 | 489 | st19061908 | Control | 1 day |
